# Unary and Binary Dynamic Column Breakthrough Experiments with Carbon Dioxide and Nitrogen Imaged by X‑ray Computed Tomography

**DOI:** 10.1021/acs.langmuir.6c00250

**Published:** 2026-05-21

**Authors:** David Büchner, Ronny Pini

**Affiliations:** Department of Chemical Engineering, 4615 Imperial College London, London SW7 2AZ, U.K.

## Abstract

This study extends the digital adsorption (DA) method—i.e., adsorption experiments augmented with X-ray computed tomography (XCT)—from unary systems with strongly adsorbing CO_2_ to unary systems with weakly adsorbing N_2_ and to binary systems involving both species. Using this method, equilibrium adsorption isotherms on commercial activated carbon and zeolite 13X were measured for both gases at 294.15 K between 0.1 and 9 bar. Our analysis shows that X-ray attenuation by the bulk phase must be considered for weak adsorbates, while it is relevant only at elevated pressures for strong adsorbates. Unary dynamic column breakthrough (DCB) experiments with N_2_ showed that, with a correction factor, the DA method can describe the transient progression of the internal adsorbed phase under near-isothermal, equilibrium-controlled conditions quantitatively. In binary CO_2_–N_2_ DCB experiments, the direct determination of adsorbed amounts requires local composition data; however, when complemented by a one-dimensional DCB model, the experimental results are shown to capture dynamic adsorption behavior, including fast N_2_ and slower CO_2_ uptake, as well as a thermally enhanced roll-up. These results demonstrate that XCT provides valuable insight into dynamic adsorption processes and that, with care, the DA method can also be applied to weakly adsorbing systems.

## Graphical abstract

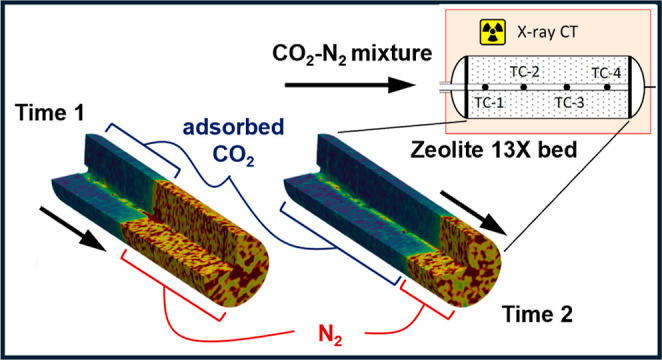

## Introduction

Adsorption-based gas separation and purification play a vital role in chemical processing and environmental sustainability.^1,2^ Among their diverse applications, carbon capture, utilization, and storage (CCUS) has emerged as a critical strategy for mitigating postcombustion CO_2_ emissions, which requires efficient separation of CO_2_ from flue gas components such as N_2_.^3–5^ Achieving such separations relies not only on the design of the adsorption process but also on the selection of appropriate adsorbent materials. A wide range of candidates—including porous carbons, metal–organic frameworks, and zeolites—has been investigated for CO_2_ capture.^6^ Among these, zeolite 13X is often used as a benchmark adsorbent for bone-dry or moisture-controlled gas streams due to its high CO_2_-N_2_ selectivity, low cost, and established commercial use in other separation processes.^7,8^

Designing and optimizing adsorption processes typically relies on dynamic column breakthrough (DCB) experiments, which provide transient outlet concentration, flow, and local temperature data, from which competitive loadings and kinetic parameters can be derived.^9–11^ However, conventional DCB analysis yields only column-averaged information, masking local effects arising from packing heterogeneities, flow maldistribution, or thermal gradients. Recent advances also show that both experimental artifacts in breakthrough measurements, such as those arising from adsorbent shaping and pressure-drop effects, and model-based factors, including temperature-dependent kinetics and isotherm selection, can systematically bias breakthrough interpretation and derived uptake values.^12,13^ These limitations are particularly significant for multicomponent systems such as CO_2_-N_2_ compositions, where both mass and heat transfer strongly influence breakthrough behavior and apparent selectivity.^14–16^ A well-known manifestation of such coupling is the roll-up phenomenon, where the outlet concentration of a weakly adsorbing species temporarily exceeds its inlet value.^17,18^ The classical, equilibrium-driven roll-up originates from the displacement of the weaker adsorbing component by a stronger one. Subsequent studies have shown that thermal effects can profoundly modify or even dominate this behavior. Li et al.^19^ reported a dual-mode roll-up in CO_2_–H_2_O systems, where a detached thermal front propagated ahead of the adsorption wave. Pirngruber et al.^20^ observed a coupled but still distinct thermal wave in CO_2_–CH_4_ mixtures, while Ahn et al.^21^ demonstrated that even modest temperature rises can amplify the magnitude of the equilibrium roll-up without producing a separate thermal front. These studies show that, although thermal and equilibrium effects are coupled, they can give rise to distinct roll-up behaviors depending on whether heat effects form an independent wave or merely enhances equilibrium displacement. Despite these insights, direct experimental observation of such phenomena inside an operating adsorption column remains challenging, as conventional breakthrough setups provide only outlet-averaged responses.

Recent experimental methods using X-ray computed tomography (XCT) have opened new opportunities to visualize adsorption phenomena within packed beds.^22^ This development has been supported by the substantial expansion and maturation of laboratory and synchrotron X-ray imaging facilities over recent decades, which has made advanced XCT approaches increasingly practical to integrate into materials-focused research.^23,24^ Building on this foundation, the digital adsorption (DA) method leverages XCT imaging to measure changes in X-ray attenuation associated with gas adsorption, enabling the measurement of spatially resolved adsorption loading and isotherms^25,26^ and the reconstruction of three-dimensional maps of microstructural material properties, such as specific surface area and micropore volume.^27^ Applied to CO_2_ adsorption on activated carbon, the DA method has demonstrated the capability to capture internal concentration profiles and dynamic wave propagation during breakthrough experiments.^28^ Despite this promise, the method has not yet been extended to other gases or multicomponent systems, where differences in adsorption strength and density changes pose additional challenges for quantitative interpretation.

The present study addresses this gap by extending the DA method to unary and binary gas systems involving N_2_ and CO_2_ on zeolite 13X and activated carbon. For weakly adsorbing species such as N_2_, the contribution of the bulk gas phase to the overall XCT signal is significant and must be accurately accounted for. Here, a correction approach is introduced to isolate the adsorbed-phase contribution and enable quantitative comparison with conventional DCB results. The methodology is then applied to explore competitive adsorption in CO_2_–N_2_ systems, providing spatially and temporally resolved insights into the interplay between equilibrium and thermal effects during DCB experiments. These results demonstrate that XCT offers a powerful means to probe the internal dynamics of complex adsorption processes, serving as a complementary diagnostic tool, advancing both experimental characterization and model validation for next-generation gas separation technologies.

## Materials and Methods

### Digital Adsorption Method

The DA method has been successfully used to measure adsorption isotherms^26^ as well as internal 1-D profiles of the adsorbed amount during DCB experiments.^28^ At this point, a brief summary of the DA method is provided, including the central equations. XCT is a well-established technology in medicine and various scientific and engineering applications. At its core lies its capability to measure localized attenuation, i.e., the linear attenuation coefficient, which is dependent on the density and atomic number of the matter. Three-dimensional maps of the local attenuation are obtained through rotating either the X-ray source and the detector array around the sample or through rotating the sample in place, while keeping the X-ray source and detector fixed. The series of two-dimensional projections from various angles is then mathematically processed to create a three-dimensional cross-sectional image of the sample. While with some X-ray scanners the output quantity is a grayscale value, or the linear attenuation coefficient directly, in a medical XCT scanner, as used in this study, the common output quantity is the so-called CT number in Hounsfield units (HU). The CT number can be understood as a precalibrated quantity, where the calibration is obtained through the application of a linear transformation of the mass attenuation coefficient, such that the CT number of air and water correspond to −1000 HU and 0 HU, respectively. Under the assumption of a constant and narrow X-ray energy spectrum above 100 kV and within a limited density range, the calibration against known fluids also allows for the approximation of the CT number as a linear function of the fluid’s mass density ρ

1 CT(ρ)=aρ+b

where *a* and *b* are fluid dependent calibration coefficients.^29^ Fundamentally, this equation suggests that the CT number of any given voxel can be obtained through linearly combining the contribution of all components, i.e., differently dense materials, to the total attenuation. In other words, by subtracting two XCT images, the change of the CT number of a given voxel can be directly correlated to a change of density in the respective voxel. This is the concept at the core of the DA method, which calculates the excess adsorbed amount by relating it to the change of density between a well-registered reference and a subsequent image taken after adsorption has occurred. The reference image is acquired prior to the adsorption experiment, with the bed in a fully regenerated condition and under a known inert atmosphere, for example, helium. In this case, the total CT number has contributions from the solid phase (adsorbent), the bulk gas phase, and the adsorbed phase. Assuming the solid phase remains unchanged in both the reference and subsequent image, its contribution cancels out when the difference image is calculated. As a result, the total change in CT number of a voxel at time *t* and located at X = [*x*, *y*, *z*] can be used to calculate an excess adsorption term *H* ^ex^(*t*, *X*, θ) with θ­(*t*, *X*) = [*p*(*t*, *X*), *T*(*t*, *X*), *y*(*t*, *X*)] defining the thermodynamic state through pressure *p*, temperature *T*, and gas-phase composition *y*, for every voxel or volume element.

2 $$H^{ex}\left(t,X;\theta \left(t,X\right)\right)=\overset{®}{CT}\left(t,X;\theta \left(t,X\right)\right)-\overset{®}{CT}\left(X;\theta^{*}\right)-\phi_{tot}\left(X\right)\left(CT_{g}\left(\theta \left(t,X\right)\right)-CT_{g}\left(\theta^{*}\right)\right)$$

The first part of the right-hand side of the equation is the difference between the CT number measured during the adsorption experiment $\overset{®}{CT}\left(t,X;\theta \left(t,X\right)\right)$ and a reference scan $\overset{®}{CT}\left(X;\theta^{*}\right)$ , acquired at a known reference thermodynamic state θ*. To obtain the change in CT number due to adsorption, the second term representing the change of CT number of the gas phase, composed of the total porosity ϕ_tot_(*X*) = ϕ_b_(*X*) + ϕ_p_(*X*), and CT number of the adsorptive gas CT_g_(θ­(*t*, *X*)) and CT number of the gas at the reference state CT_g_(θ*), is subtracted. Subtracting this term effectively emulates the corrections for free space and buoyancy that are applied in conventional volumetric and gravimetric measurement systems, respectively. ϕ_b_ and ϕ_p_ refer to the bed’s and the particle’s porosity, respectively. The excess in CT number can then be transferred into an excess adsorbed mass per unit volume

3 $$m^{ex}\left(t,X;\theta \left(t,X\right)\right)=H^{ex}\left(t,X;\theta \left(t,X\right)\right)/a\left(\theta \left(t,X\right)\right)$$

where *a*(θ­(*t*, *X*)) is the previously introduced calibration coefficient. Further, the excess adsorbed amount η^ex^ in moles adsorbed per kilogram of adsorbent can be obtained via the expression

4 $$\eta^{ex}\left(t,X;\theta \left(t,X\right)\right)=\frac{m^{ex}\left(t,X;\theta \left(t,X\right)\right)}{\rho_{bed}\left(X\right)M_{m}\left(\theta \left(t,X\right)\right)}$$

with ρ_bed_(*X*) being the bulk density of the bed and *M*_m_(θ­(*t*, *X*)) the effective molar mass of the adsorbate.

### Digital Adsorption Method—Considerations for Static and Dynamic Measurements with Single- and Multi-Component Systems

As of now, the DA method has only been applied to systems with CO_2_ as a single adsorptive gas, using pure helium at atmospheric conditions as the reference state.^26,28^ In static isotherm measurements employing the DA method, as performed by Joss et al.,^26^ the bulk gas phase is in equilibrium throughout the bed. Under these conditions, the thermodynamic state becomes independent of time and spatial location, such that θ­(*t*, *X*) = θ_eq_ where θ_eq_ denotes the equilibrium adsorption state. Furthermore, since equilibrium in the gas phase, and consequently in the adsorbed phase, requires temporal invariance, the expression for the excess CT number simplifies to

5 $$H^{ex}\left(X;\theta_{eq}\right)=\overset{®}{CT}\left(X;\theta_{eq}\right)-\overset{®}{CT}\left(X;\theta^{*}\right)-\phi_{tot}\left(X\right)\left(CT_{g}\left(\theta_{eq}\right)-CT_{g}\left(\theta^{*}\right)\right)$$

The CT number at adsorption equilibrium, $\overset{®}{CT}\left(X;\theta_{eq}\right)$ , and at the reference state, $\overset{®}{CT}\left(X;\theta^{*}\right)$ , can be measured directly as absolute values using XCT. Similarly, the local total porosity ϕ_tot_(*X*) can either be measured through XCT^25^ or an average porosity measured and assumed uniform across the bed. The gas-phase CT numbers at adsorption equilibrium, CT_g_(θ_eq_), and at the reference state, CT_g_(θ*) can be determined through the respective calibration measurements. Consequently, in this static formulation, all quantities appearing in eq 5 can be explicitly determined. In case of a single adsorptive component, the effective molar mass in eq 4 reduces to the molar mass of this component. This enables accurate measurements of unary excess adsorption isotherms using the DA method.^26^

Using eq 5, the excess in CT number can be directly extended to any system with multiple adsorptive gases (multicomponent system). The gas-phase CT numbers at equilibrium, CT_g_(θ_eq_), must then be obtained through XCT measurements of the respective mixture, or, assuming ideal gas behavior and linear density–CT response, they can be estimated as

6 $$CT_{g}=\sum_{i}\omega_{i}CT_{g,i}$$

with the mass fraction ω_*i*_ and gas CT number CT_g,*i*_ of (pure) component *i*. Further, under the assumption of a linear density–CT response, the calibration curves eq 1 are expected to collapse onto a single linear curve, with *a* ≈ 1.^26,29^ Therefore, a total excess adsorbed mass can be calculated through eq 3. Because it does not discriminate between the individual components in the mixture, this total excess adsorbed mass would correspond to the mass measured with traditional gravimetric adsorption isotherm measurements of mixtures.

In a DCB experiment, the bed is not in thermodynamic and adsorption equilibrium until full breakthrough is achieved. The temperature and concentration of the gas phase, as well as the amount, composition, and temperature of the adsorbed phase, vary with time and location. As a result, eq 2 can not be simplified further. While both $\overset{®}{CT}\left(t,X;\theta \left(t,X\right)\right)$ and $\overset{®}{CT}\left(X;\theta^{*}\right)$ can still be measured in absolute terms, the calculation of the CT number of the bulk gas phase CT_g_(θ­(*t*, *X*)) would require knowledge of its local and instantaneous composition. This information is not directly accessible in a continuous way through experimental measurements. Pini et al.^28^ suggest that CT_g_(θ­(*t*, *X*)) ≈ CT_g_(θ*) if the DCB experiment is conducted at the same temperature and pressure as the reference state. This assumption allows removing the contribution of the bulk gas phase to the overall CT number change in eq 2. Under these conditions, the evolution of the total excess adsorbed amount can be estimated in both single- and multicomponent DCB experiments.

### Digital Adsorption Method—Bulk Gas Correction

Neglecting the effect of gas phase composition on the total CT number change, as done in Pini et al.,^28^ has been shown to yield accurate results for DCB experiments involving a single, strongly adsorbing gas such as CO_2_. However, as demonstrated in the Results section of this study, this assumption is no longer valid for weakly adsorbing gases, for which the bulk gas phase contribution becomes non-negligible and must be explicitly accounted for. To address this limitation, a general correction approach is proposed here. To this end, we reformulate the bulk gas term in eq 2 as

7 $$\phi_{tot}\left(X\right)\left(CT_{g}\left(\theta \left(t,X\right)\right)-CT_{g}\left(\theta^{*}\right)\right)\approx S\left(t,X\right)\phi_{tot}\left(X\right)\left(CT_{g}\left(\theta_{eq}\right)-CT_{g}\left(\theta^{*}\right)\right)$$

where *S*(*t*, *X*) is the local correction factor, and CT_g_(θ_eq_) the CT number of the gas phase after equilibrium has been achieved, i.e., the state corresponding to the inlet feed composition in a DCB experiment. The term (CT_g_(θ_eq_) – CT_g_(θ^*^)) is therefore a constant value which is scaled to mimic the transient change of the bulk gas concentration throughout the bed. The correction factor is computed as

8 $$S\left(t,X\right)=\frac{\overset{®}{CT}\left(t,X;\theta \left(t,X\right)\right)-\overset{®}{CT}\left(X;\theta^{*}\right)}{\overset{®}{CT}\left(X;\theta_{eq}\right)-\overset{®}{CT}\left(X;\theta^{*}\right)}$$

and is defined such that *S* = 0 when $\overset{®}{CT}\left(t,X;\theta \left(t,X\right)\right)=\overset{®}{CT}\left(X;\theta^{*}\right)$ (no adsorption) and *S* = 1 when $\overset{®}{CT}\left(t,X;\theta \left(t,X\right)\right)=\overset{®}{CT}\left(X;\theta_{eq}\right)$ (equilibrium achieved). Between these limits, *S*(*t*, *X*) interpolates linearly. While this method should, in principle, be applicable to single- and multicomponent systems, it still relies on the assumption that the bulk-gas phase concentration front moves with the same velocity and exhibits the same dispersion as the adsorbed-phase.

### Experimental Setups

The experimental setup used for the isotherm measurements is shown in Figure 1a. The central part of the custom-made column is a PEEK tube (Ensinger Ltd., TECAPEEK natural), housing the adsorbent bed. The length, inner, and outer diameters of the packed bed are 273 mm, 30 mm, and 50 mm, respectively. PEEK was selected because of its beneficial radiolucent properties, which prevent imaging artifacts and thereby enable more accurate imaging results. At both ends of the tube, a small recess accommodates two disks, one sieve-like and one with a conical diameter increase, designed to ensure proper confinement of the bed and optimized gas distribution. Both disks are securely pressed into the PEEK tube using custom-made caps, which also serve as connection points for peripheral sensors and gas supply lines. This design ensures a tight seal while maintaining optimal gas flow and diffusion into the bed. The caps have an additional round and oval elongation, used to locate them in a custom-made column holder, which in turn is horizontally fixed onto the scanner bed. The pressure was measured using two pressure transmitters (Keller UK, PA-23SY), and one thermocouple (TC Ltd., Ultrafine wired K type) which was attached to the outside of the column about 50 mm into the bed. Pressure and temperature are continuously recorded with a data logger (Pico Technology Ltd., TC-08). A vacuum pump was attached to the outlet side of the column to obtain pressures below 1 bar. Isotherms were measured on two microporous adsorbent materials, zeolite 13X rods (Sigma-Aldrich, diameter ≈1.6 mm, batch MKCS3238) and activated carbon rods (Sigma-Aldrich, Norit RB3, diameter ≈3 mm, batch BCCJ6516). The feed gases were supplied by BOC at their respective CP grade, Helium 99.999%, CO_2_ 99.995%, and N_2_ 99.9992%.

**Figure 1:**
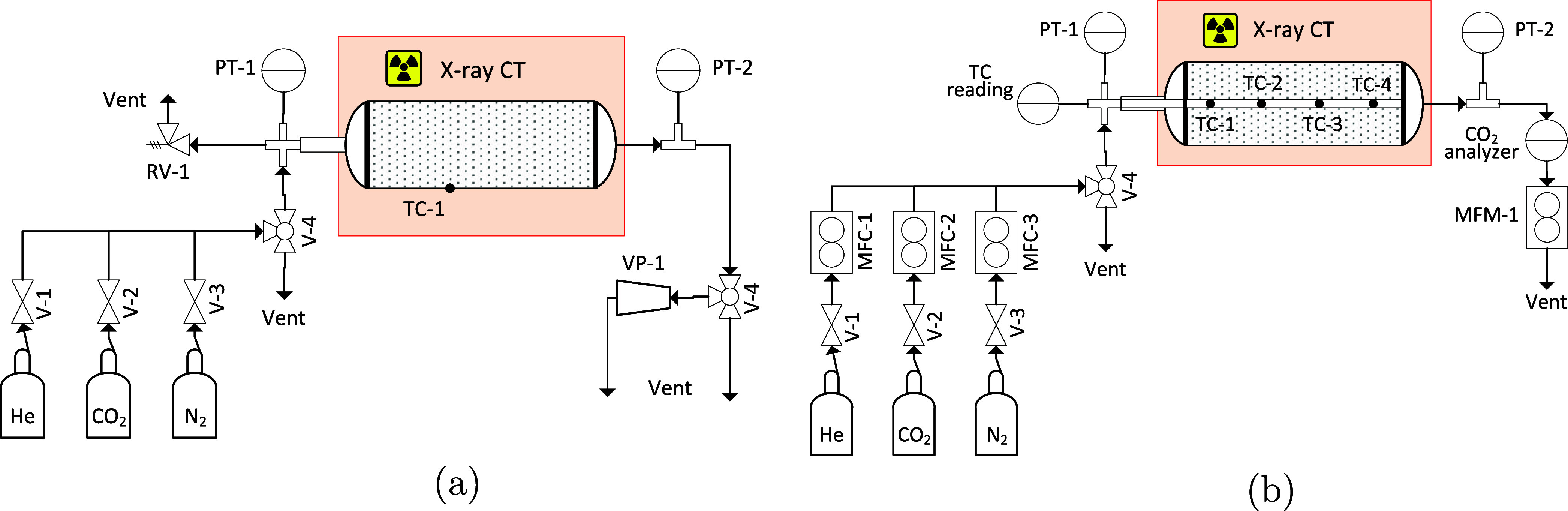
Flow-diagrams of the experimental setup used for the adsorption isotherm measurments (a) and dynamic column breakthrough experiments (b). *V* valve, RV relief valve, PT pressure transducer, VP vacuum pump, MFC mass flow controller, MFM mass flow meter, TC thermocouple.

Figure 1b depicts the experimental setup used to conduct the DCB experiments. The same adsorption column, pressure transmitter, and data logger, as in the previously described setup for the isotherm measurements, were used. Four thermocouples (TC Ltd., Ultrafine wired K type) were routed axially along the bed center, inside a polyethylene tube (3.2 mm outer diameter). At about 1/8, 3/8, 5/8, and 7/8 of the bed length *L*, respectively, the thermocouple nodes were moved through a hole out of the tube and fixed to its outside. The exact locations of the thermocouple junctions with respect to the bed inlet were determined through visual inspection of the X-ray images for each experiment. The inlet flow rate was prescribed using a mass flow controller (Alicat Scientific, MC-500SCCM-D), while the outlet flow conditions were recorded through one CO_2_ analyzer (Gas Sensing Solutions Ltd., SprintIR-R) and one mass flow meter (Alicat Scientific, M-1 SLPM-D). The three DCB experiments were conducted on zeolite 13X rods (Sigma-Aldrich, diameter ≈1.6 mm, batch MKCS3238) using the same CP grade gases purchased from BOC.

### Experimental Procedure: Isotherm Measurements

Four separate isotherm measurement experiments, one with each adsorbent material and gas, were conducted at ambient temperature (approximately 294.15 K) using dry adsorbents. The activated carbon rods were regenerated overnight at 423.15 K, while the zeolite 13X rods were regenerated at 563.15 K, to ensure the removal of moisture and any potential contaminants. Following regeneration, the adsorbents were packed into the vertically placed PEEK column. A continuous flow of helium was maintained throughout the packing process to avoid exposure to air. Between the four experiments, the column was thoroughly emptied and cleaned to prevent cross-contamination between the adsorbents.

After packing, the column was positioned in the column holder, which was premounted and aligned to the scanner bed. A controlled helium atmosphere at 2 bar was established, and two initial scans, referred to as “reference scans”, were acquired. Subsequently, the column was not moved for the entire experiment.

The gas adsorption isotherm measurements were performed in two distinct modes: adsorption and desorption. During the adsorption mode, the outlet side of the column was sealed, and the pressure was incrementally increased to 1, 3, 5, 7, and 9 bar. The pressure was controlled using a gas pressure regulator, and after each adjustment and an initial adsorption period of 15 min, the inlet was closed. The system was then allowed to equilibrate until the pressure and temperature had stabilized for a minimum of 30 min. Upon reaching equilibrium, two X-ray scans, referred to as “adsorption scans”, were acquired. This procedure was repeated for each pressure step.

During the desorption phase, the inlet side of the column remained sealed, and pressure was gradually reduced to 2, 1, 0.8, 0.6, 0.4, and 0.2 bar by opening the outlet valve to a vacuum pump. Once the desired pressure was achieved, the outlet valve was closed, and the system, similar to the experiments in adsorption mode, was allowed to equilibrate, i.e., a stabilization of pressure and temperature was observed for at least 30 min. Subsequently, two “desorption scans” were acquired. This process was repeated for each pressure step during desorption.

X-ray imaging was conducted using a Toshiba Aquilon 64 Scanner set to a tube voltage of 120 kV and a current of 200 mA. Images were reconstructed with an *x* × *y* × *z* resolution of 0.117 × 0.117 × 1 mm, without the application of additional filtering. The complete length of the packed bed was scanned, with a field of view in the *x*–*y* plane of 59.9 mm. The scanning time for a complete scan was approximately 10 s. After completion of the measurements, the adsorbent was removed from the column, regenerated under the same conditions as prior to the experiment, and weighted using a laboratory scale. The bed volume of 1.929 × 10^–4^ ± 1.29 × 10^–6^ m^3^ and measured mass *m* was used to compute the bulk density of the bed ρ_bed_ and subsequently the average total porosity, defined as ϕ_tot,avg_ = 1 – ρ_bed_/ρ_s_, where ρ_s_ is the skeletal density of the adsorbent, which was assumed from literature. Since pore volume is not used in the modeling or interpretation in this study, no further pore-structure characterization was required. The calculated values are summarized in Table 1. During the postprocessing, a cylindrical section, with a diameter corresponding to the column diameter and length of 100 mm, was segmented to determine the adsorbed amount. The total time for completing one full adsorption isotherm measurement across all pressure points was approximately 12 h. Image analysis and data processing were performed using custom Python scripts developed in-house.

**Table 1:** Material and Bed Properties of the Adsorption Isotherm Measurements

parameter	zeolite 13X	activated carbon
*m* [kg]	0.121 ± 0.001	0.087 ± 0.001
ρ_bed_ [kg/m^3^]	627.3 ± 6.673	450.8 ± 6.0
ρ_s_ [kg/m^3^]	2567 ± 40^a^	2160 ± 340^a^
ϕ_tot,avg_ [-]	0.7557 ± 0.01426	0.7913 ± 0.1250
CT_s_ [HU]	2222 ± 189	984 ± 1193

a Value taken from the literature.^26^

### Experimental Procedure: Dynamic Column Breakthrough Experiments

Three DCB experiments were conducted at ambient temperature (approximately 294.15 K) and into the ambient atmosphere (approximately 0.99 bar). After regenerating the zeolite 13X rods at 563.15 K overnight, the adsorbent was packed into the vertically placed PEEK column while a helium purge was maintained. Each experiment was performed on a freshly regenerated and newly packed bed of the same zeolite 13X rods.

After packing each bed, the column was moved into the preinstalled column holder, and a helium purge (100 sccm, standard cubic centimeters per minute, referenced to 25 °C and 1 atm) was maintained until the temperature at all of the four thermocouple nodes had converged to and remained at room temperature for at least 1 h. After the equilibrated state of the bed was obtained, two repeated X-ray scans, referred to as the “reference scan”, were acquired. Subsequently, at time *t* = 0 s, the DCB experiment was initiated through the application of a step change in the inlet flow gas composition. For the three DCB experiments, the total mass flow rate was 100 sccm, while the inlet gas composition varied (unary CO_2_, unary N_2_, and a binary mixture of 50 vol % CO_2_ and 50 vol % N_2_).

The system delay was corrected by measuring the gas holdup volumes upstream and downstream of the packed bed using repeated isopropanol displacement measurements, yielding 9.6 cm^3^ and 9.4 cm^3^, respectively. The overall dead volume of 19 cm^3^, therefore, corresponds to 8.9% of the system volume, which is well below the upper limit of approximately 20–30%, beyond which dead-volume effects begin to significantly distort the breakthrough front shape and residence-time interpretation.^9^ For the present CO_2_ on zeolite 13X system, which is strongly adsorbing, transient reductions in the outlet volumetric flow can in principle amplify downstream dead-volume effects.^30,31^ However, the detector was located closely downstream of the packed bed and the downstream dead volume was limited to 9.4 cm^3^, ensuring that dead-volume effects on the measured breakthrough response remained limited. At a volumetric flow rate of 100 sccm, the inlet volume corresponds to a dead time of 5.76 s, which was used to temporally align the DCB with the corresponding XCT measurements.

Accordingly, the first CT scan was acquired at *t* = 0 s, corresponding to 5.76 s after the initiation of the DCB experiment. In the case of the unary CO_2_ and N_2_ experiments, subsequent scans were taken at 300 and 60 s intervals, respectively. After the initial breakthrough was observed through an increase of the outlet flow rate, the lengths of the scanning intervals were partially increased. During the binary CO_2_-N_2_ experiment, the initial scanning interval was reduced to 45 s, necessary to track the fast-moving N_2_ front, and was then increased to 900 s until the first CO_2_ breakthrough was observed. The DCB experiments were considered complete after the outlet flow rate was equal to the inlet flow rate, and all four temperature measurements had converged to room temperature.

### DCB Modeling

A one-dimensional DCB model, developed and described previously by Ward et al.,^32^ was employed to simulate the transient adsorption experiments. The model simulates the behavior of a compressible, nonisothermal gas mixture containing three components: CO_2_, N_2_, and He, with He acting as an inert, nonadsorbing component. The pressure drop along the column is calculated using Darcy’s law, and the gas and solid phases are assumed to remain in thermal equilibrium throughout the column. Mass transfer into the solid phase is represented by a linear driving force (LDF) model under macropore diffusion control. The associated intraparticle diffusion rate constant was calculated through $k=\frac{15\phi_{p}D_{m}}{\tau_{p}{r_{p}}^{2}}$ , with the particle porosity ϕ*_p_*, molecular diffusivity *D*_m_, and particle tortuosity τ_*p*_ assumed from literature and summarized in Table 2. The core of the model comprises coupled mass and energy balances, including overall and component mass balances for the fluid phase, component mass balances for the solid phase, and energy balances for both the column and wall. These balances account for advection and longitudinal dispersion in the gas phase, as well as heat released by adsorption and heat exchange between the gas, column wall, and surroundings, through conduction and convection. The Supporting Information file provides the corresponding partial differential equations and boundary conditions (Section 1). The equations are discretized in space using a semi-implicit finite-volume scheme. Time integration for the unary simulations was performed using MATLAB’s ode15s solver with the mass-matrix approach, while for the binary case, ode23t was found to be more efficient and therefore used.

**Table 2:** Physical Parameters and Properties of the DCB Model

parameter	value	units
Geometric dimensions		
column length, *L*	0.273	[m]
inner column diameter, *D*_in_	0.03	[m]
outer column diameter, *D*_out_	0.05	[m]
thermocouple diameter, *D*_TC_	0.0032	[m]
Feed conditions		
feed pressure, *p*_F_	100,000	[Pa]
feed temperature, *T*_F_	294.15	[K]
Ambient conditions		
ambient temperature, *T*_∞_	294.15	[K]
Physical properties of bed/adsorbent		
particle porosity, ϕ_p_	0.5751	[-]
particle diameter, *D*_p_	1.6 × 10^–3^	[m]
particle tortuosity, τ_p_	3	[-]
molecular diffusivity, *D*_m_	1.5 × 10^–5^	[m^2^ s^–1^]
heat capacity of adsorbent, *C*_p,s_	1070	[J kg^–1^ K^–1^]
thermal conductivity of adsorbent, *k*_s_	0.63	[J m^–1^ K^–1^ s^–1^]
Physical properties of gas/adsorbate CO_2_ N_2_ CO_2_-N_2_		
molar mass of gas, *M*_m,g_	0.044 0.028	[kg mol^–1^]
density of gas, ρ_g_ × 10^5^	1.87 1.249 1.594	[kg m^–3^]
dynamic viscosity of gas, μ_g_ × 10^5^	1.48 1.76 1.6	[kg m^–1^ s^–1^]
heat capacity of gas, *C*_p,g_	846 1040 921	[J kg^–1^ K^–1^]
heat capacity of adsorbate, *C*_p,a_	846 1040 921	[J kg^–1^ K^–1^]
thermal conductivity of gas, *k*_g_	0.0168 0.026 0.0211	[J m^–1^ K^–1^ s^–1^]
effective thermal conductivity, *k*_*z*_	0.0474 0.070 0.05813	[J m^–1^ K^–1^ s^–1^]
Physical properties of column wall		
density of column wall, ρ_W_	627.3	[kg m^–3^]
heat capacity of column wall, *C*_p,W_	1100	[J kg^–1^ K^–1^]
thermal conductivity of column wall, *k*_W_	0.27	[J m^–1^ K^–1^ s^–1^]

All parameters describing the physical properties of the modeled system are summarized in Tables 1 and 2. To reflect the decreased cross-sectional area due to the rod housing of the thermocouples, an adjusted equivalent diameter $D_{e}=2\sqrt{{\left(D_{in}/2\right)}^{2}-{\left(D_{TC}/2\right)}^{2}}$ was used as the inner column diameter in the simulation. At the given conditions, and in contrast to Ward et al.^32^ who used a stainless steel column, the model was found to be sensitive to both inside and outside heat transfer coefficients. Accordingly, both were fitted to obtain the optimal match of the model solution for each of the three DCB experiments. The optimizations were carried out using MATLAB’s genetic algorithm function *ga*, with the following cost function to be minimized for the optimization parameter *h*_in_ (inner heat transfer coefficient) and *h*_out_ (outer heat transfer coefficient).

9 $$J_{T}\left(h_{out},h_{in}\right)=\frac{1}{n_{TC}n_{p,T}}\sum_{j=1}^{n_{TC}}\sum_{i=1}^{n_{p,T}}{\left(T_{TC,j}^{sim}\left(h_{out},h_{in},t_{i}\right)-T_{TC,j}^{\exp}\left(t_{i}\right)\right)}^{2}$$

The cost function *J*_T_ represents the mean squared error between the experimentally measured temperatures, *T*_TC,j_^exp^, and the corresponding simulated temperatures, *T*_TC,j_^sim^. The summation is carried out over all measurement times *t*_*i*_ for each thermocouple *j*, with a total of *n*_p,T_ time points and *n*_TC_ thermocouples distributed along the axial positions. As multiple and sufficiently high local temperature readings were obtained, it was not considered necessary to include the flow rate response in the cost function.

## Results and Discussion

### CT Number Change due to Adsorption

As part of the isotherm measurements using the DA method, four sets of 3D XCT scans at respective total pressures were acquired—one set for CO_2_ and N_2_ adsorption, and for activated carbon and zeolite 13X, respectively. Since the isotherm measurements were performed at constant temperature and with pure adsorptive gases, the thermodynamic state θ reduces to a single variable, namely the pressure *p*, in the following analysis. As can be seen in Table 3, the average total difference in CT number $\overset{®}{CT}\left(p_{eq}\right)-\overset{®}{CT}\left(p^{*}\right)$ ranges from 0.5148 HU and 0.5946 HU at 0.1 bar for N_2_ adsorption on activated carbon and zeolite 13X, respectively, to 133.0 HU and 152.9 HU at 9 bar for CO_2_ adsorption on the same adsorbents. Calibration measurements were conducted to obtain the explicit relationship CT_g_(*ρ*) = *aρ* + *b* for each adsorptive. The parameters *a* and *b* were fitted for He, CO_2_, and N_2_ measurements, respectively, and are reported in the Supporting Information (Section 2). The calculated bulk gas contribution during the adsorption experiments −ϕ_tot_(CT_g_(*p*_eq_) – CT_g_(*p* ^*^)) is provided for selected pressures in the right column of Table 3 (the value in parentheses refers to % contribution to the value $\overset{®}{CT}\left(p_{eq}\right)-\overset{®}{CT}\left(p^{*}\right)$ ). When comparing this relative share *R*(*p*), it is apparent from Table 3 and more clearly Figure 2, that different behaviors are observed for the CO_2_ and N_2_ data sets. For CO_2_ adsorption, *R*(*p*) increases nonlinearly but monotonically, rising from below 1% at vacuum pressures to just under 10% at 9 bar, for both adsorbents. In contrast, for N_2_ adsorption, *R*(*p*) decreases from above 25% at low vacuum pressures, reaches a local minimum of about 17% near 1 bar, and then increases similarly to the CO_2_ adsorption curve, to just above 25% at 9 bar, for both adsorbents.

**Table 3:** Excess CT Attenuation from Static Unary Adsorption Experiments

	*H* ^ex^	$\overset{®}{CT}\left(p_{eq}\right)-\overset{®}{CT}\left(p^{*}\right)$	–ϕ_tot_(*CT*_g_(*p*_eq_) – *CT*_g_(*p* ^*^))
Activated carbon CO_2_			
0.1 bar	11.0401	11.1866	0.1466 (1.3%)
1 bar	43.9251	45.3844	1.4593 (3.2%)
9 bar	121.123	133.0229	11.8999 (8.9%)
Activated carbon N_2_			
0.1 bar	0.3463	0.5148	–0.1686 (26%)
1 bar	4.5429	5.4728	–0.9298 (17.3%)
9 bar	20.5357	27.95	–7.4143 (25.7%)
Zeolite 13X CO_2_			
0.1 bar	75.2359	75.3931	0.1572 (0.2%)
1 bar	114.7692	116.1269	1.3577 (1.2%)
9 bar	141.3743	152.9365	11.5622 (7.6%)
Zeolite 13X N_2_			
0.1 bar	0.4402	0.5949	0.1546 (32.8%)
1 bar	3.9844	4.8652	0.8808 (17%)
9 bar	20.8139	28.0074	7.1935 (26.5%)

**Figure 2:**
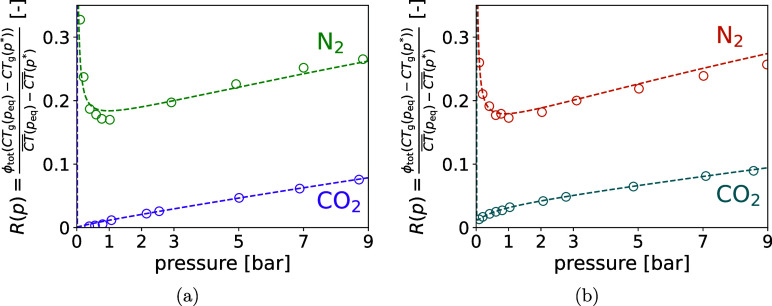
Ratio $R\left(p\right)=\phi_{tot}\left(CT_{g}\left(p_{eq}\right)-CT_{g}\left(p^{*}\right)\right)/\left(\overset{®}{CT}\left(p_{eq}\right)-\overset{®}{CT}\left(p^{*}\right)\right)$ as a function of pressure, *p*_eq_, for zeolite 13X (a) and activated carbon (b). Circles correspond to the measured values, and the dashed lines to the explicit analytical solution derived from the DSL isotherm parametrization.

### Relative Contribution of Bulk Gas to CT Number Changes

The contrasting behavior of *R*(*p*) for CO_2_ and N_2_ adsorption shown in Figure 2 arises from the relative magnitudes and growth rates of the CT number contributions from adsorption and the bulk gas phase. As shown in Table 3, the total CT change $\overset{®}{CT}\left(p_{eq}\right)-\overset{®}{CT}\left(p^{*}\right)$ is substantially larger for CO_2_ than for N_2_, reaching over 150 HU at 9 bar compared to only a few tens of HU for N_2_. In this system, CT_g_(p*) is constant, while CT_g_(*p*_eq_) increases linearly with pressure. Consequently, the bulk gas contribution −ϕ_tot_(*X*)­(*CT*_g_(*p*_eq_) – *CT*_g_(*p* ^*^)) is linear with a slope that can be exactly calculated from the total porosity and gas properties.

For CO_2_, this linear bulk gas contribution is small compared to the total CT change, producing a monotonic, slowly increasing *R*(*p*) from below 1% to roughly 10%. For N_2_, the bulk gas contribution is comparable to the total CT change at low pressures, giving initial *R*(*p*) values above 25%. As adsorption increasingly contributes to the total CT signal, the relative share of the bulk gas decreases, creating a local minimum near 1 bar. At higher pressures, the total CT change due to adsorption continues to increase but begins to saturate, i.e., its slope decreases, while the bulk gas contribution remains linear with constant slope. Consequently, the numerator slope exceeds that of the denominator, causing *R*(*p*) to increase again, eventually approaching the same trend as for CO_2_. From a mathematical point of view, this behavior can be explained by expressing the relative contribution as

10 $$A\left(p\right)=\overset{®}{CT}\left(p_{eq}\right)-\overset{®}{CT}\left(p^{*}\right),,B\left(p\right)=-\phi_{tot}\left(X\right)\left(CT_{g}\left(p_{eq}\right)-CT_{g}\left(p^{*}\right)\right)$$

where *R*(*p*) = *B*(*p*)/*A*(*p*). The observed minimum in *R*(*p*) occurs where the relative growth rate of the total CT change equals that of the bulk gas contribution

11 $$\frac{d}{dp}\ln A\left(p\right)=\frac{1}{B\left(p\right)}\frac{dB}{dp}$$

which provides a compact analytical condition for the pressure *p*_min_ at which *R*(*p*) reaches its minimum. Through this formulation, the pressure *p*_min_ can be determined numerically from the measured total CT change. Alternatively, if the excess CT change *H* ^ex^(*p*) = *A*(*p*) + *B*(*p*) is expressed via an adsorption isotherm model such as the DSL model, *p*_min_ can be calculated explicitly from the known model parameters, providing a direct connection between the adsorption behavior and the observed minimum in the relative bulk-gas contribution. The complete derivations and the explicit formulation to calculate *p*_min_ are provided in the Supporting Information (Section 6).

The explicit stationary condition was solved numerically using the Newton method. For N_2_ adsorption, analytical solutions were obtained, yielding *p*_min_ ≈ 96.3 kPa for zeolite 13X and *p*_min_ ≈ 71.3 kPa for activated carbon, in excellent agreement with the minima observed in the measured *R*(*p*) curves shown in Figure 2. In contrast, no solution was found for CO_2_ adsorption on either adsorbent, consistent with the monotonic increase of *R*(*p*) in the experimental data.

Overall, for CO_2_ adsorption, the relative bulk-gas contribution *R*(*p*) increases monotonically but remains very small at typical measurement pressures (e.g., 3.2% for activated carbon and 1.2% for zeolite 13X at 1 bar), allowing the bulk-gas contribution to be safely neglected in the total CT number change, as done in previous studies.^28^ In contrast, N_2_ adsorption exhibits a nonmonotonic behavior in *R*(*p*), with a distinct minimum at intermediate pressures and substantially higher contributions—roughly an order of magnitude larger than for CO_2_. While Joss et al.^26^ argue that the bulk-gas term may be neglected at low pressures, this approximation is only robust for strongly adsorbing systems. For nitrogen and other weakly adsorbing systems, the bulk-gas contribution remains significant relative to the adsorption signal and must therefore be explicitly included when deriving excess adsorption from CT number changes using the DA method.

### Equilibrium Adsorption Isotherms Measurements

Equations 2–4 were applied to obtain the molar excess adsorbed amounts per unit mass of adsorbed at each isotherm point. Figure 3 shows the measured isotherm as empty circles, with the error bars expressing the respective uncertainty, i.e., one standard deviation. Uncertainties were determined through the classic rules of image noise calculation and error propagation as described in detail in the Supporting Information (Section 3–5).

**Figure 3:**
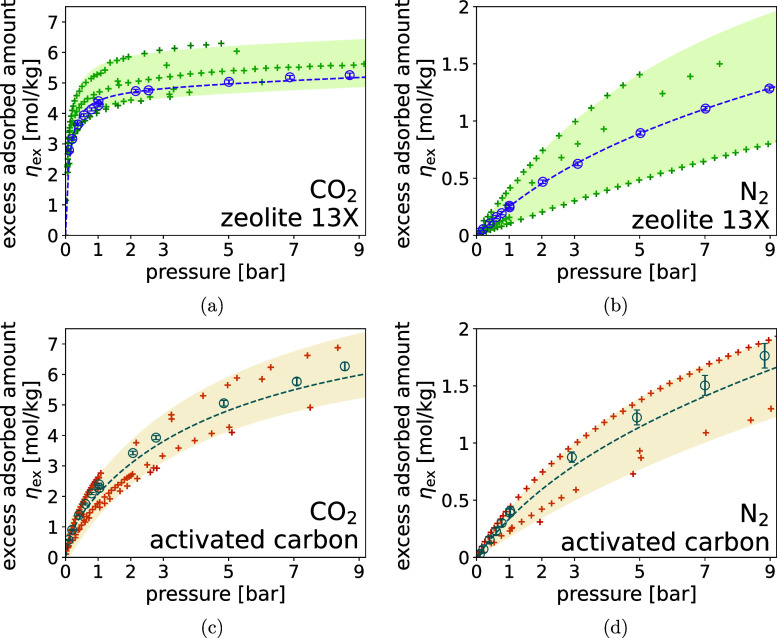
Equilibrium excess adsorption isotherms of CO_2_ and N_2_ on zeolite 13X, (a,b), and activated carbon (c,d) at 294.15 K, respectively. Excess adsorption amounts have been calculated within a 100 mm thick section of the adsorbent column, equivalent to a total volume of roughly 70 685 mm^2^ or total mass of 44.42 and 31.87 g of zeolite 13X and AC, respectively. Selected literature data at similar temperatures (±4 K) is for zeolite 13X^14,26,33–36^ and activated carbon^26,28,33,37–41^ indicated by the crosses.

The isotherm measurements with their respective uncertainties are also provided in tabular form in the Supporting Information (Section 7). The uncertainty associated with the CO_2_ isotherms remains fairly constant and below 2% and 4% for zeolite 13X and activated carbon, respectively. As reported in Joss et al.,^26^ the error on the estimated bed density, which propagates through into the total bed porosity, is dominating the overall uncertainty. This error is the same for CO_2_ and N_2_ adsorption. However, as the excess amount adsorbed is smaller in the case of N_2_ adsorption on both adsorbent materials, the relative uncertainty of the N_2_ isotherms is higher. It ranges from 52% and 92% at the lowest vacuum pressure, to 2% and 6% at high pressures, for zeolite 13X and activated carbon, respectively.

All four isotherms exhibit the characteristic features of a type I adsorption isotherm. The CO_2_ isotherms, in particular, illustrate the distinction between an adsorbent with a predominantly uniform micropore structure and strong ion–quadrupole interactions, such as zeolite 13X, and one where a broader pore size distribution, together with dispersion-driven physisorption, governs adsorption, as is typical for activated carbons. The CO_2_ isotherm of zeolite 13X displays a steep uptake at low pressures, reaching 2.8 mol·kg^–1^ at 0.1 bar, followed by an early saturation plateau at higher pressures. In contrast, the CO_2_ isotherm of activated carbon increases more gradually and continues to rise appreciably over the entire pressure range. While the N_2_ isotherms on both adsorbents also exhibit a convex type I shape, the curvature is less pronounced than for CO_2_, approaching a more linear behavior over the investigated pressure range. The equilibrium capacities are considerably lower, reaching only 1.3 mol·kg^–1^ on zeolite 13X and 1.8 mol·kg^–1^ on activated carbon at 9 bar. This behavior reflects the weaker interactions of N_2_ with the adsorbent surface of zeolite 13X. As a result, the uptake on zeolite 13X is strongly suppressed compared to CO_2_, whereas activated carbon, with its broader distribution of micro- and meso-pores, accommodates N_2_ more effectively and thus exhibits a higher overall capacity.

To obtain explicit formulations of the adsorption isotherms, the dual-site Langmuir (DSL) model was employed for both CO_2_ and N_2_ isotherms. The DSL model is required to account for the stronger adsorption affinity and the presence of distinct adsorption sites arising from the heterogeneous pore structure of the adsorbents.^42^ The DSL isotherm model is described through

12 $$q^{*}=\frac{q_{s1}b_{1}c}{1+b_{1}c}+\frac{q_{s2}b_{2}c}{1+b_{2}c}$$

with the equilibrium absolute amount adsorbed q* expressed as a function of concentration *c*, and the temperature independent saturation capacities *q*_*s*1_ and *q*_*s*2_ and adsorption equilibrium constants *b*_1_ and *b*_2_ for each of the two sites, respectively. The adsorption equilibrium constant for each site *i* is temperature dependent and follows the van’t Hoff equation

13 $$b_{i}=b_{0,i}\;\exp\left(-\Delta U_{i}/RT\right)$$

where *b*_0,*i*_ is the reference equilibrium constant, and Δ*U*_*i*_ the change of internal energy due to adsorption. Assuming ideal gas behavior (*c* = *p*/*RT*), the DSL isotherm model is directly expressed as a function of partial pressure instead of molar concentration. Typically, the six parameters *q*_*s*1_, *q*_*s*2_, *b*_0,1_, *b*_0,2_, Δ*U*_1_, and Δ*U*_2_ are fitted to experimental data of at least four isotherms. However, as in this work, isotherms could only be measured at one specified temperature (room temperature), it is not meaningful to fit the change of internal energy Δ*U*_*i*_. Instead, from literature, typical values were fixed,^28,32,41^ and the remaining four parameters fitted against the CO_2_ isotherm measurements. For thermodynamic consistency, the sum of the saturation capacities was then fixed for the respective adsorbent, $q_{s1,CO_{2}}+q_{s2,CO_{2}}=q_{s1,N_{2}}+q_{s2,N_{2}}$ . Subsequently, ω, *b*_0,1_ and *b*_0,2_ were fitted against the N_2_ isotherm measurements, whereby *q*_*s*1,N_2__ = ω·*q*_*s*1,CO_2__ and *q*_*s*2,N_2__ = (1 – ω)·*q*_*s*1,CO_2__. The fitting was conducted using the curve fit function of the scipy Python package, which employs a nonlinear least-squares method. It is noted that while the DA method measures excess adsorbed amount, the DSL model defines an absolute adsorbed amount. As this work focuses on the lower-pressure region, the difference between the two is assumed negligible. The fitted isotherms parameters are summarized in Table 4 and the resulting isotherms at measurement conditions are shown as dashed lines in Figure 3. The four fitted isotherm show an overall good agreement with the measured excess adsorbent amounts. In the case of the CO_2_ and N_2_ isotherms on the zeolite 13X, the model prediction is well within the estimated uncertainties of the measurements. This is mostly also true for the N_2_ isotherm on activated carbon, while for the CO_2_ isotherm, a consistent under-prediction of the excess adsorbed amount is observed.

**Table 4:** Langmuir Isotherm Parameter

adsorbent	parameter	CO_2_	N_2_
zeolite 13X	*q*_*s*1_ [mol/kg]	2.98	2.309
	*q*_*s*2_ [mol/kg]	2.444	3.115
	*b*_0,1_ [m^3^/mol]	3.588 × 10^–7^	1.13 × 10^–6^
	*b*_0,2_ [m^3^/mol]	1.31 × 10^–8^	5.26 × 10^–8^
	Δ*U*_1_ [J/mol]	–36 600^a^	–18 981^b^
	Δ*U*_2_ [J/mol]	–35 700^a^	–18 582^b^
activated carbon	*q*_*s*1_ [mol/kg]	0.88	7.647
	*q*_*s*2_ [mol/kg]	8.041	1.274
	*b*_0,1_ [m^3^/mol]	1.822 × 10^–6^	7.924 × 10^–7^
	*b*_0,2_ [m^3^/mol]	1.297 × 10^–6^	4.994 × 10^–6^
	Δ*U*_1_ [J/mol]	–28 630^c^	–14 807^b^
	Δ*U*_2_ [J/mol]	–20 370^c^	–17 501^b^

a From literature Ward et al. 2022.^32^

b From literature Park et al. 2019.^41^

c From literature Pini et al. 2021.^28^

### Comparison of CO_2_ and N_2_ Adsorption Isotherms

The DA method provides reliable adsorption measurements across a range of pressures and adsorbates. For CO_2_, it yields low uncertainties on both zeolite 13X and activated carbon, demonstrating good reproducibility and consistency with prior work using the DA approach.^26^ For N_2_, the relatively small excess uptakes at low pressures naturally lead to larger relative uncertainties, reflecting the intrinsic challenges of accurately quantifying weakly adsorbing gases.^43,44^ Other studies on these adsorbents, using conventional volumetric or gravimetric techniques, further support the expected adsorption behavior and variability.^45,46^ Overall, these results highlight that the DA method can produce meaningful and reproducible adsorption data while addressing the experimental challenges inherent to weakly interacting adsorbates.

The observed shapes of the CO_2_ and N_2_ isotherms align well with the structural characteristics of the adsorbents. Zeolite 13X, with its uniform micropore structure, exhibits the steep initial uptake and early saturation typical of type I behavior,^45^ whereas activated carbon, possessing a broader and more evenly distributed range of micro- and mesopores, shows a more gradual adsorption increase across the pressure range.^46^ In addition to the excess adsorption isotherms measured with the DA method, Figure 3 shows various isotherms at similar temperatures (294.15 ± 4 K) measured with volumetric and gravimetric methods for the same adsorbent materials. For zeolite 13X, the literature data set used for comparison consists entirely of binder-formed samples, including spherical beads,^14,26,35^ cylindrical beads,^33^ and extruded pellets.^34^ The activated carbons included (Chemviron,^37^ BPL 3 × 10,^33^ AC, 2GA-H2J,^41^ Norit R1,^39^ RB2 ^38^ and RB3 ^26,28,40^ ) are also commercial formed materials. Both the CO_2_ and N_2_ isotherms obtained in this study fall well within the range covered by literature data, which is expected given the normal variation in adsorption capacity among binder-formed zeolite 13X samples and between different commercial activated carbons. While Joss et al.^26^ demonstrated the capability of the DA method to resolve CO_2_ isotherms on zeolite 13X and activated carbon at pressures above 1 bar, this work successfully extends the method’s applicability to vacuum pressures and the more weakly adsorbing N_2_.

It should be noted that all measurements were performed at a single temperature, and the internal energy of adsorption was assumed from literature values.^28,32,37^ Conducting adsorption experiments at multiple temperatures would provide a more rigorous determination of thermodynamic properties and further validate the DA method, particularly for weakly interacting gases.

### DCB Experiments: Conventional Output and Model Fitting

In total, three DCB experiments were conducted following the previously described procedure. Figure 4 shows the conventionally measured DCB output—transient outlet flow rates and temperatures at the specified locations—for unary CO_2_ (a,b), unary N_2_ (c,d), and a 50 vol % CO_2_–50 vol % N_2_ mixture (e,f), respectively. The experimental measurements are represented by the red full line in the flow rate response, and the colored square symbols in the temperature plots. Time is nondimensionalised using the residence time *t*_r_ = *V*_bed_/*f*_in_, where *V*_bed_ is the volume of the empty column (m^3^) and *f*_in_ the inlet flow rate (m^3^/s). Since the inlet flow rate was identical in all three experiments, the reference time is the same as well in all three experiments (*t*_r_ = 135.6 s). For the two unary DCB experiments, the model results were optimized by fitting the inner and outer heat transfer coefficients to the first and fourth temperature measurements, using the defined cost function, eq 9. These two were chosen as they provided the most consistent data, whereas including all four measurements was found to overconstrain the optimization. For the binary experiment, the optimization was based on the first and third temperature measurements, as the fourth exhibited an inconsistency shortly before the onset of the large temperature spike. The obtained optimal heat transfer coefficients are summarized in Table 5. The evaluation of the cost function, i.e. mean squared error in temperature, for the optimal pair of heat transfer coefficients yields *J*_T_ = 1.176 K^2^, 0.01991 K^2^, and 2.424 K^2^ for the unary CO_2_, unary N_2_, and binary CO_2_–N_2_ DCB experiments, respectively. Considering the outside heat transfer coefficients, the value obtained for the unary CO_2_ DCB experiment is within or near the upper end of the range expected for natural convection and minimal forced convection in a laboratory environment.^47,48^ However, the value of 28.2 W·m^–2^ and 26.26 W·m^–2^ obtained for the unary N_2_ and binary CO_2_–N_2_ experiment, respectively, lie well outside this physically plausible range. This indicates that they likely reflect parameter compensation in the model and possible inaccuracies in temperature sensing rather than a true physical external heat transfer coefficient. The inside heat transfer coefficient is not readily approximated through physical correlations; however, the obtained values are within the range of inside heat transfer coefficients used in CO_2_ DCB models in literature.^28,32^

**Figure 4:**
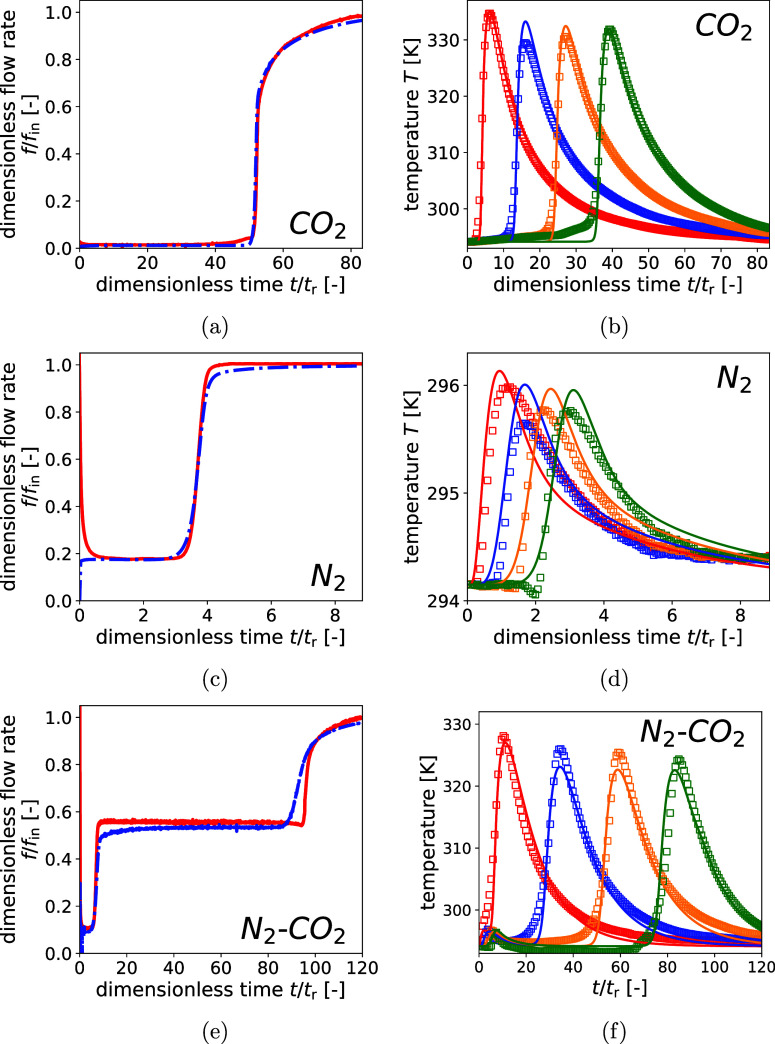
Flow rate and temperature measurements from three dynamic column breakthrough experiments with 100 sccm CO_2_ (top row), 100 sccm N_2_ (middle row), and a binary CO_2_-N_2_ mixture at 50/50 with a total flow rate of 100 sccm (bottom row). Experimental and model outlet flow rates are represented by solid red and dashed blue lines, respectively. Temperatures at approximately one, three, five, and seven-eighths of the column length *L* are shown in red, blue, yellow, and green, respectively (experimental data as square symbols; model predictions as solid lines).

**Table 5:** Fitted Inside and Outside Heat Coefficients

CO_2_ flow rate [sccm]	N_2_ flow rate [sccm]	*h*_out_ W/m^2^ K	*h*_in_ W/m^2^ K
100	-	9.248	15.77
-	100	27.99	53.30
50	50	23.13	8.171

In Figure 4, the model results are shown as blue dashed lines for the flow rate response and as solid colored lines for the temperature profiles. In the two binary experiments, peak temperatures recorded by the second and third thermocouples were lower than those of the first and fourth, in contrast to the gradual decrease predicted by the model. The consistent relative differences between peaks suggest imperfect calibration of the middle thermocouples. However, since the absolute deviations are small and only the outer thermocouple data were used for model fitting, this is acceptable within the scope of this work. The flow rate response curves show excellent agreement in breakthrough time and shape between experiments and model predictions for both binary cases, confirming the validity of fitting heat transfer coefficients using multiple temperature measurements. A noteworthy deviation is a small increase in flow rate just before the sudden jump, observed in the CO_2_ DCB experiment. This coincides with a slight temperature rise at the last thermocouple prior to the sharp increase. This premature breakthrough suggests minor gas channeling, which can occur near the wall where the bed density is reduced.^49^ The ratio of the column to particle diameter (18.75) is close to 20, which is considered sufficient to minimize near-wall effects,^50^ and above 10, the minimum value recommended by Wilkins et al.^9^ However, these thresholds do not account for a solid rod (thermocouples) along the center of the bed, which is reducing the bed density further, and thus promotes channeling, despite the sufficient bed to particle diameter radius.

The model prediction for the binary CO_2_–N_2_ DCB experiment shows overall good agreement regarding the location and magnitude of the slower temperature front caused by CO_2_ adsorption. The model predicts a faster propagation of the sharp temperature front than observed experimentally, with peak temperatures occurring increasingly ahead of the measurements over time. Additionally, the simulated temperature rise is more abrupt, whereas the experimental data show a slower, gradual increase before the sharp jump. A plot of the temperature profiles with a restricted time axis up to N_2_ breakthrough, provided in the Supporting Information file (Section 8), shows that the model significantly underpredicts the temperature rise from N_2_ adsorption at the three downstream thermocouples. At the first thermocouple, the model additionally predicts a temporary temperature drop between the local N_2_ peak and the onset of CO_2_ adsorption, whereas the experiments show a continued increase with a temporarily lower gradient. The mismatch before N_2_ breakthrough is attributed to the small temperature rise during N_2_ adsorption and its short duration relative to the overall experiment. Consequently, the cost function used for model fitting is dominated by the larger temperature increase during CO_2_ adsorption, causing the model to underpredict the N_2_-related temperature changes.

With regard to the conventional flow rate output, for this experiment, absolute and component flow rates could not be determined through calibration, as only the CO_2_ concentration was measured, and the composition of the CO_2_–N_2_–He mixture is therefore unknown. Instead, the experimental flow rate was normalized by the absolute flow rate measured at the end of the experiment. The flow rate response shows good agreement with regard to N_2_ breakthrough time. The intermediate plateau observed between N_2_ and CO_2_ breakthrough is slightly higher in the experimental measurements than in the model, which can be attributed to the noncalibrated measurements. A clear difference between experimental measurements and model predictions is present in the CO_2_ breakthrough response. The model predicts a considerably earlier initial breakthrough and a more dispersed breakthrough curve. Only when converging to the input flow rate do the curves start to overlap well again. This difference is in line with the previous observation of faster advancing temperature peaks in the model.

### Unary DCB Experiments: Spatially Resolved Internal Amount Adsorbed

Next to the conventional DCB measurements, the DA method was employed to measure the internal amount adsorbed at regular time intervals. Figure 5a shows 13 1-dimensional profiles of the amount adsorbed measured throughout the unary CO_2_ DCB experiment. Each circular symbol represents the amount adsorbed averaged over a 2 mm slice of the bed. As demonstrated in a similar experiment by Pini et al.,^28^ the shape of the internal profiles closely resembles that of the previously introduced flow rate breakthrough curve. Furthermore, the color scheme clearly illustrates the advancement of the adsorbed CO_2_ front through the bed over the course of the experiment. Discarding the first and last 10 mm of the bed, the average amount adsorbed at the close of the experiment is 4.138 mol/kg, which is slightly below the 4.266 mol/kg measured during the DA isotherm measurements at the same conditions. The sliced-averaged adsorbed amount has a standard deviation of 0.0517 mol/kg, which matches the estimated method’s uncertainty of 0.0517 mol/kg. However, the observed maximum deviation of ±0.161 mol/kg is ≈3.1× the standard deviation and far exceeds the method’s uncertainty, indicating that these variations likely arise from heterogeneities inherent in random packings, as previously suggested by Pini et al.^28^ As solid lines, the figure also shows the internal profiles of the adsorbed amount of CO_2_ obtained from the model fitted to the temperature measurements. Comparison with the experimental profiles at the same relative time indicates great consistency. However, the model-predicted front of the adsorbed phase initially lags slightly behind the experimentally measured one, before propagating at a higher velocity and eventually surpassing the experimental profiles. This deviation is likely attributable to the thickness effect within the packing—specifically, a locally reduced bulk density near the inlet and outlet^51,52^ —which in the experiments facilitates a more rapid penetration speed of the front and may also reflect small inaccuracies introduced through temperature-based parameter fitting.

**Figure 5:**
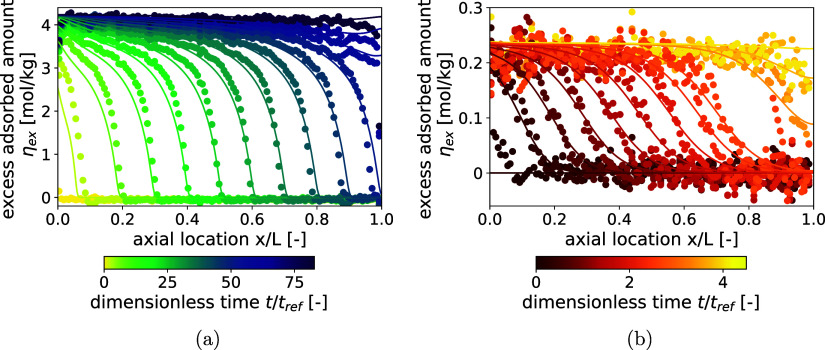
Internal adsorbed amount profiles obtained through the application of the DA method to the unary CO_2_ (a) and N_2_ (b) DCB experiments. Symbols indicate the experimental values averaged over a 2 mm section along the bed axis, and the solid curves show the DCB model predictions derived from parameters fitted to the conventional temperature measurements. Times are represented by the color code with respect to the residence time.

Figure 5b shows the progression of the adsorbed-phase front during the unary N_2_ DCB experiment. The excess adsorbed amount was calculated from the XCT images using the DA method with the previously introduced correction factor. The average CT number at equilibrium, $\overset{®}{CT}\left(X;\theta_{eq}\right)$ , and the CT number difference $\overset{®}{CT}\left(X;\theta_{eq}\right)-\overset{®}{CT}\left(X;\theta^{*}\right)$ at τ = 4.5 are −210.6 HU and 5.0 HU, respectively. Assuming complete exchange of He with N_2_ in the bulk phase, the CT number contribution CT_g_(θ_eq_) – CT_g_(θ*) was calculated to be 1.153 HU, based on the CT number gas calibration measurements provided in the Supporting Information (Section 2). The one-dimensional profiles clearly show that introducing the correction factor produces the expected behavior; that is, no significant N_2_ adsorption occurs ahead of the adsorbed-phase front, while at saturation the level of excess adsorbed amount agrees well with the values obtained from the DA isotherm measurements. The average adsorbed amount at saturation in the N_2_ DCB experiment is 0.2206 mol/kg, slightly lower than the value obtained from the isotherm measurements under identical conditions (0.2458 mol/kg), which marginally exceeds the estimated method’s uncertainty (0.0144 mol/kg). This difference can be attributed to the presence of thermocouple tubing in the column, which reduces the effective adsorbent mass while the same bed density is applied, leading to a slight underestimation of the calculated adsorbed amount. The maximum deviation and standard deviation for N_2_ are ±0.0716 mol/kg and 0.0156 mol/kg, respectively, both notably smaller than the corresponding values for CO_2_. This supports the interpretation that the observed deviations primarily arise from inherent heterogeneities in the local bed density. Since the total amount of N_2_ adsorbed is considerably smaller than that of CO_2_, these density variations produce a proportionally weaker amplification of the adsorbed-phase fluctuations. The internal profiles at corresponding times obtained from the model are again depicted as solid lines in the Figure. For the N_2_ DCB experiment, the predicted one-dimensional profiles of the adsorbed amount are in good agreement with the experimental data, consistent with the unary CO_2_ DCB case. A minor deviation is observed at later relative times: while the experimental profiles show that the adsorbed-phase front remains relatively constant in shape, the model predicts an increasingly dispersed front, leading to a gentler slope.

### DCB Behavior of Weakly Adsorbing Components

As shown by the preceding results, the change in bulk gas composition contributes substantially to the overall variation in CT number for weakly adsorbing systems. In the case of nitrogen, the contribution from the bulk gas phase was found to be at least 17%. While this effect can be readily accounted for in static isotherm measurements using the DA method, it cannot be directly quantified in DCB experiments, where the local composition of the bulk gas phase is difficult to measure experimentally. To address this limitation, the present study introduces a correction factor that incorporates the CT number variation associated with changes in the bulk gas phase. This correction exploits the linear mixing behavior of X-ray attenuation in low-Z gas mixtures, for which the CT number scales linearly with bulk gas density and composition.

In the present formulation, the scaling factor S further assumes that variations in bulk gas composition scale linearly with the local adsorbed amount, which strictly holds only for linear adsorption isotherms. The validity of this assumption, in the case of the here studied unary N_2_ DCB experiment, is supported by the internal profiles of the relative amount adsorbed and the mole fraction of nitrogen at discrete times obtained with the DCB model (shown in the Supporting Information, Section 8), which exhibit an almost perfect overlap. This observation indicates that the conducted experiment was equilibrium-controlled and operated near isothermal conditions.^11^ The good agreement between the internal profiles obtained through the DA method and the model therefore demonstrates that, with the introduction of the correction factor, the DA method can also be applied to study the internal dynamics of more weakly adsorbing systems, provided that the system is equilibrium-controlled, near isothermal, and exhibits a near linear adsorption isotherm. For systems exhibiting strongly nonlinear isotherms, the definition of the scaling factor could, in principle, be improved by explicitly incorporating the corresponding isotherm parametrization.

### Breakthrough Curve Reconstruction from Internal Adsorption Profiles

The internal adsorbed amount at given times can further be used as an alternative way to obtain the conventional breakthrough curve. For this purpose, a molar balance is calculated to obtain the molar outlet flow rate

14 $${ṅ}_{out}\left(t_{i}\right)={ṅ}_{in}-{ṅ}_{ads}\left(t_{i}\right)$$

at time *t*_*i*_, where ${ṅ}_{in}$ is the molar inlet flow rate, and ${ṅ}_{ads}\left(t_{i}\right)$ the molar adsorption rate. The instantaneous adsorption rate can be calculated through

15 $${ṅ}_{ads}\left(t_{i}\right)=\frac{m_{b}\left({\mathrm{\eta ̅}}^{ex}\left(t_{i}\right)-{\mathrm{\eta ̅}}^{ex}\left(t_{i-1}\right)\right)}{t_{i}-t_{i-1}}$$

were ${\mathrm{\eta ̅}}^{ex}$ is the total amount adsorbed in the bed, and *m*_b_ the total mass of adsorbent in the bed. The conversion between volumetric flow rate *f* in sccm and molar flow rate ṅ in mol s^–1^ is carried out assuming ideal-gas behavior at standard conditions (298.15 K and 1 atm), yielding $ṅ=6.83\times 10^{-7}f$ . Figure 6 shows the breakthrough curve obtained through molar balance calculations, utilizing the DA measurements of the unary CO_2_ DCB experiment. Although the experimental data results in somewhat oscillating values, it can be seen that the molar balance approach reproduces the breakthrough curve accurately. The breakthrough time is in excellent agreement with the time predicted by the model, and the tail of the breakthrough curve is well captured. However, because of the low sampling rate, i.e., the long time between two consecutive XCT scans, the initial sharp rise of the flow rate at breakthrough is only captured through two measurement points. For the same reason, no meaningful curve was obtained through this approach when applied to the unary N_2_ DCB experiment. In this case, the scanning interval is too large in relation to the overall duration of the experiment, i.e., too few points are available to reproduce the breakthrough curve.

**Figure 6:**
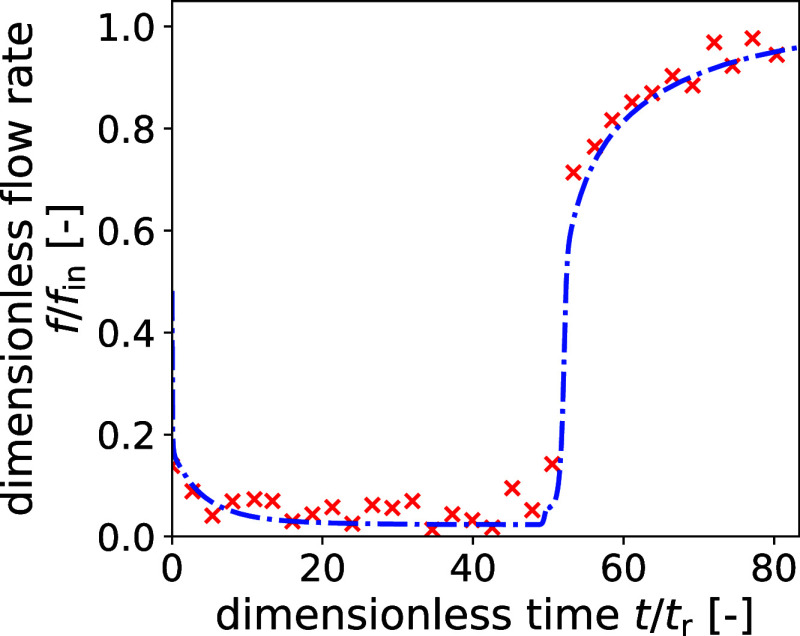
Breakthrough curve of the unary CO_2_ DCB experiment, calculated through the molar balance approach. The red crosses and the dashed blue line represent the experimental and model data, respectively.

### Binary DCB Experiment: Spatially Resolved Internal Profiles

The correction factor method could in principle, also be applied to the binary CO_2_-N_2_ DCB experiment to calculate a total (excess) adsorbed mass via the DA method. However, this would require additional considerations of the bulk gas phase contribution, because of the development of multiple transitions, i.e., from the reference state to a pure N_2_ composition (to yield a difference of 1.153 HU) and to a 50/50 CO_2_-N_2_ composition (to yield an additional difference of 0.313 HU). These two stages would need to be reflected in the correction factor, which could be defined to have an intermediate state. Alternatively, as it has been done in the unary CO_2_ DCB experiment, the second smaller change to the CO_2_-N_2_ mixture could be neglected. However, for clarity the correction factor has not been applied to the binary experiment and the local absolute change in CT number $\Delta \overset{®}{CT}=\overset{®}{CT}\left(t,X;\theta \left(t,X\right)\right)-\overset{®}{CT}\left(X;\theta^{*}\right)$ -and thus density— is reported instead. For eight discrete time steps, i.e., XCT scans acquired throughout the binary DCB experiment, Figure 7 shows a 3-dimensional visualization of the packed bed, with the left upper quarter of the cylindrical cross-section cut out. The multisequential color code, applied to the local CT number difference, $\Delta \overset{®}{CT}$ , was defined such that the two stages of the experiment are clearly distinguishable. Starting from a regenerated bed at *t*/*t*_r_ = 0.4, a first change in CT number (transitioning from dark red to yellow-red grains) is observed, progressing continuously through the bed and corresponding to the fast-moving N_2_ adsorption front. This front reaches the end of the column at *t*/*t*_r_ = 7.7, in agreement with the N_2_ breakthrough time determined from the measured response in the gas flow rate. Subsequently, the slower-moving CO_2_-rich adsorption front (dark blue-green grains) advances through the bed until it reaches the column end at *t*/*t*_r_ = 94.1, which likewise agrees well with the experimentally observed time of initial CO_2_ breakthrough, i.e. the second step increase in flow rate seen in Figure 4e. The thermocouple array is clearly visible as the region of no to low CT number difference along the core of the packed bed.

**Figure 7:**
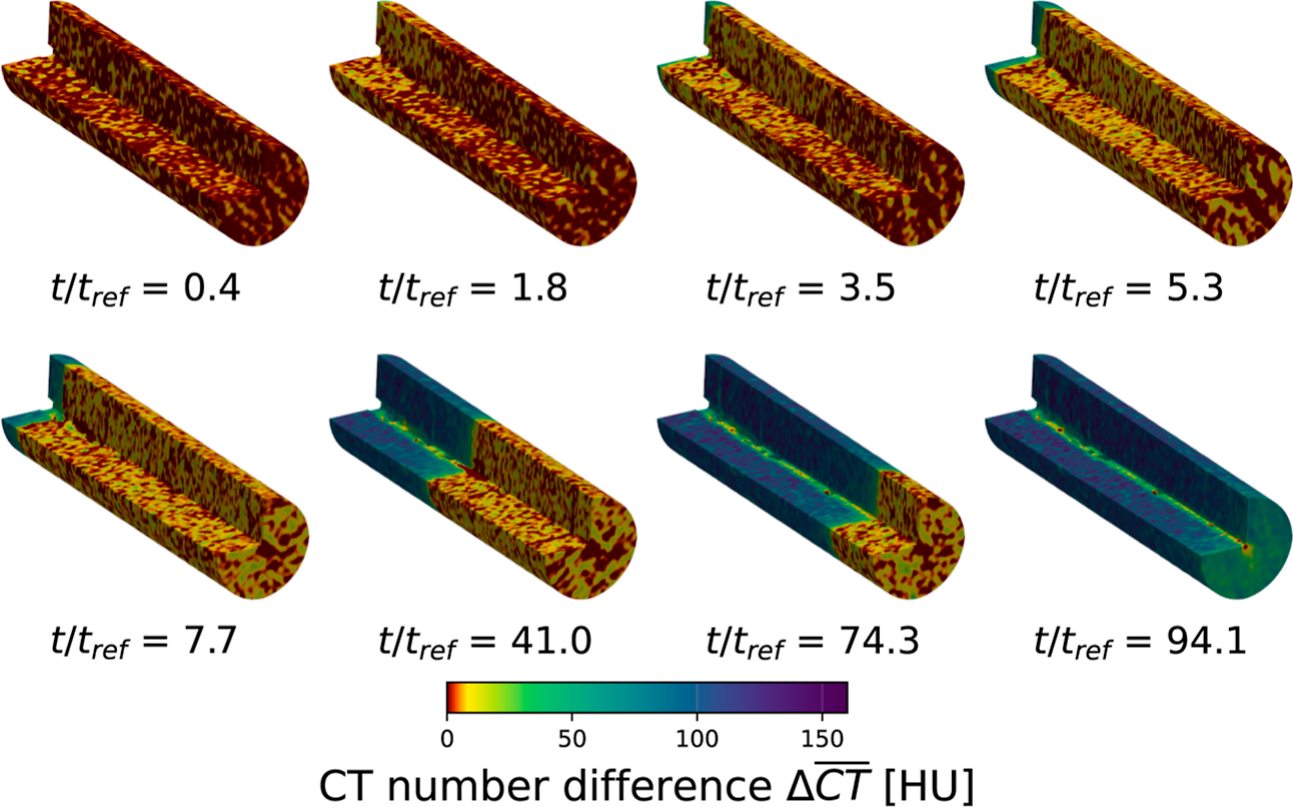
Three-dimensional visualization of the local CT number differences $\Delta \overset{®}{CT}=\overset{®}{CT}\left(t,X;\theta \left(t,X\right)\right)-\overset{®}{CT}\left(X;\theta^{*}\right)$ at different relative times during the CO_2_-N_2_ DCB experiment. The flow direction is from the upper left to the lower right end of the bed. For better visualization, the column axis is scaled by 1/2 and the voxel resolution convoluted with a 2 × 2 × 2 mm kernel.

To facilitate a quantitative analysis of the binary DCB experiment, one-dimensional profiles of the slice-averaged CT number difference $\Delta \overset{®}{CT}$ are shown in Figure 8a. A simple 4 mm moving average convolution was applied to reduce the imaging noise while also preserving relevant details. The color code to indicate the relative time was again chosen in such a way that the time until N_2_ breakthrough and from N_2_ to the breakthrough of the feed gas mixture are captured by a transition from dark red to yellow, and yellow to dark purple, respectively. The logarithmic scaling of the *y*-axis promotes the clear recognition of the two phases of the binary DCB experiment. In the first phase, which is associated with the time until N_2_ breaks through, the CT number difference continuously increases from 0 HU to 4.469 HU. Assuming that only N_2_ has been adsorbed and that helium in the bulk gas phase has been completely replaced by nitrogen, application of the DA method yields an adsorbed amount of 0.222 mol/kg. This value is in good agreement with those obtained during the unary DCB experiment and from the isotherm measurements. In the second phase of the experiment, the CT number difference increases further to a considerably higher level of 99.85 HU. This increase is associated with the adsorption of CO_2_. The DCB model predicts a CO_2_ mole fraction of 99.9% in the adsorbed phase after complete breakthrough, supporting the assumption of a pure CO_2_ adsorbed phase. Under these conditions, and for a bulk gas phase with a 50/50 N_2_–CO_2_ composition, the DA method yields an excess adsorbed amount of CO_2_ of 3.671 mol/kg. This value lies between the previously measured equilibrium adsorbed amounts 3.637 mol/kg and 3.940 mol/kg, corresponding to partial CO_2_ pressures of 0.38 and 0.56 bar, respectively. The minor reduction can likely be attributed to the reduced cross-sectional area as well as wall effects around the TC, which reduce the packed bed density and therefore adsorption capacity.

**Figure 8:**
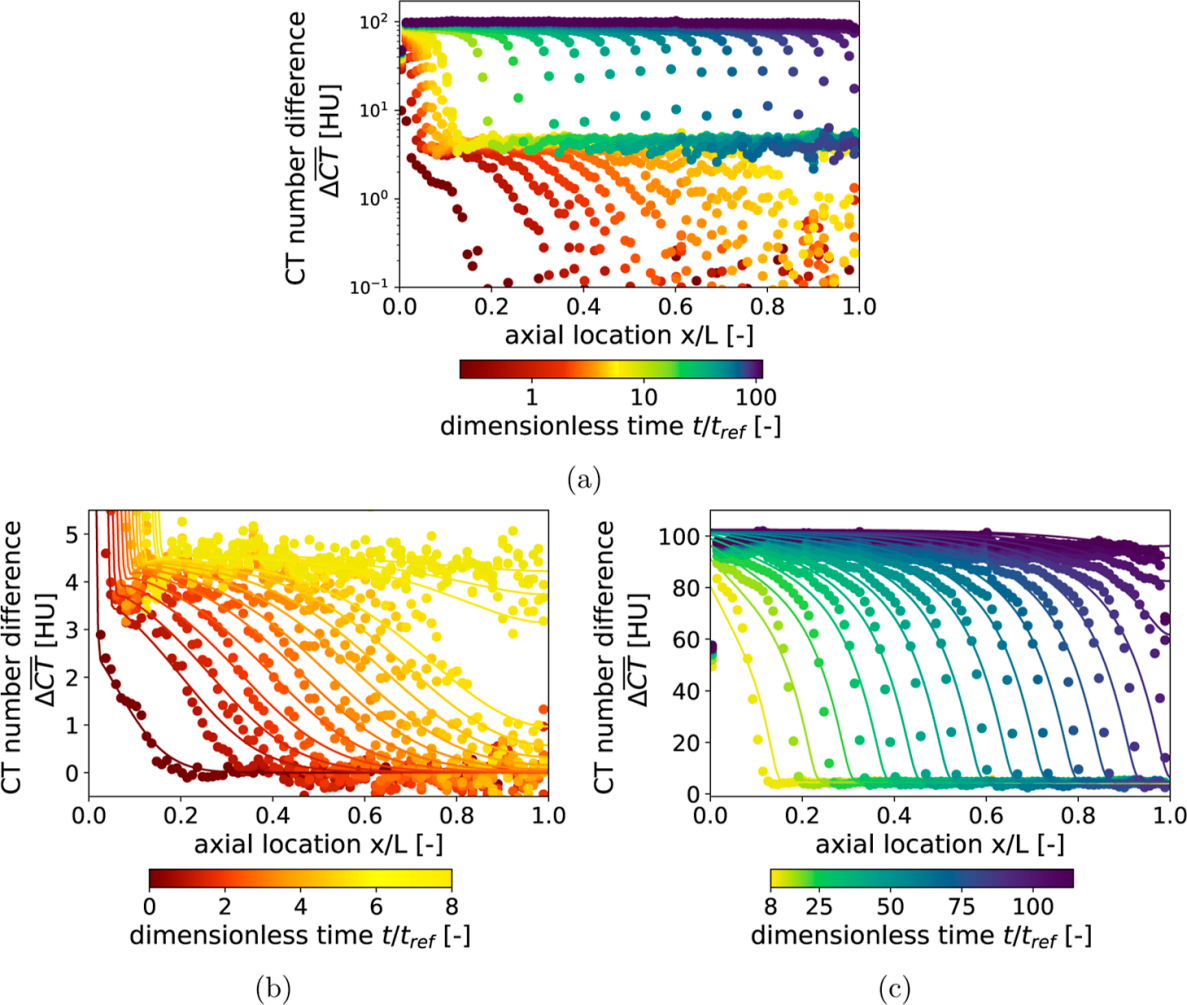
(a) Full CT number difference range; (b) range corresponding to the fast-moving N_2_ front; and (c) range corresponding to the slow-moving CO_2_-rich front. Experimental data (symbols) were smoothed using a 4 mm moving-average convolution along the bed axis. Solid curves in (b,c) represent DCB model predictions based on parameters fitted to conventional temperature measurements. Times are represented by the color code with respect to the residence time of the total flow rate.

Figure 8b,c present the same internal profiles, with the *y*-axes scaled linearly and cropped to the ranges corresponding to N_2_ and CO_2_ adsorption, respectively. The CT number difference $\Delta \overset{®}{CT}$ was reverse-calculated from the output of the model calibrated for the binary DCB experiment to enable a quantitative comparison with the experiments. The local and instantaneous adsorbed amounts of N_2_ and CO_2_ were individually converted into excess CT numbers using eqs 4 and 3 and summed up to obtain *H* ^ex^(*t*, *X*; θ­(*t*, *X*)). The CT number change associated with variations in the gas-phase composition CT_g_(θ_eq_) – CT_g_(θ*) was determined using the local and instantaneous composition of the gas phase, the gas–CT number calibrations reported in the Supporting Information (Section 2), and the gas mixture CT number definition, (eq 6). Using the local CT number changes from adsorption and gas-phase composition and applying the rearranged eq 2, yielded the model-predicted total CT number change, which is shown as solid lines in the figures for each relative time. The model accurately reproduces the CT number difference of the N_2_ plateau (4.61 HU) and the final equilibrium state after CO_2_ breakthrough (101.2 HU). During the first phase [Figure 8b], the profiles reconstructed from the model output are flatter than the experimental ones, indicating a less dispersed N_2_ front in the experiment. In the second phase [Figure 8c], the model captured the overall shape of the profiles somewhat better, although the initial increase was less steep. As the profiles approached saturation, agreement with the experimental data improved. The increase in CT number difference predicted by the model occurred earlier than observed experimentally. This suggests a faster and more dispersed CO_2_ propagation through the bed in the simulation, which is supported by the deviation of the flow rate response curve.

The binary CO_2_–N_2_ DCB experiment therefore highlights the ability of XCT to capture complex adsorption dynamics. Although the DA method cannot be applied to resolve the two adsorbing components independently, since this would require knowledge of the local and instantaneous composition of the adsorbed phase, direct analysis of the CT number differences can still be interpreted. The sequential N_2_ and CO_2_ fronts are clearly resolved, and the CT number changes provide a powerful and effective means to visualize and interpret dynamic behavior in the adsorbed phase throughout the DCB experiment.

Next to capturing the two steps of the binary DCB experiments, the one-dimensional profiles of the CT number difference also reveal an intermediate reduction of the CT number difference, as seen in Figure 9a. The profiles are only shown for four, i.e., every third, relative times to not clutter the figure and highlight the intermediate reductions. While some variation is observed around the mean CT number difference of the plateau (4.469 HU; standard deviation: 0.2801 HU), the observed dip clearly exceeds this variability and increases with relative time. To quantify this behavior, Figure 9b presents the minimum value of the dip—averaged over a 3 mm thick slice centered at the local minimum—relative to the plateau mean. A distinct trend is evident, with the relative reduction in CT number difference increasing from approximately 10% to 35% over time. Profiles of the total CT number difference reconstructed from the model output, indicated by the red line in Figure 9c, show a comparable reduction, though it is notably smaller and decreases marginally from around 2.1% to 1.4% throughout the second phase of the experiment. The CT number difference of the bulk gas phase, relative to the regenerated state, increases only minimally when N_2_ is displaced by CO_2_.

**Figure 9:**
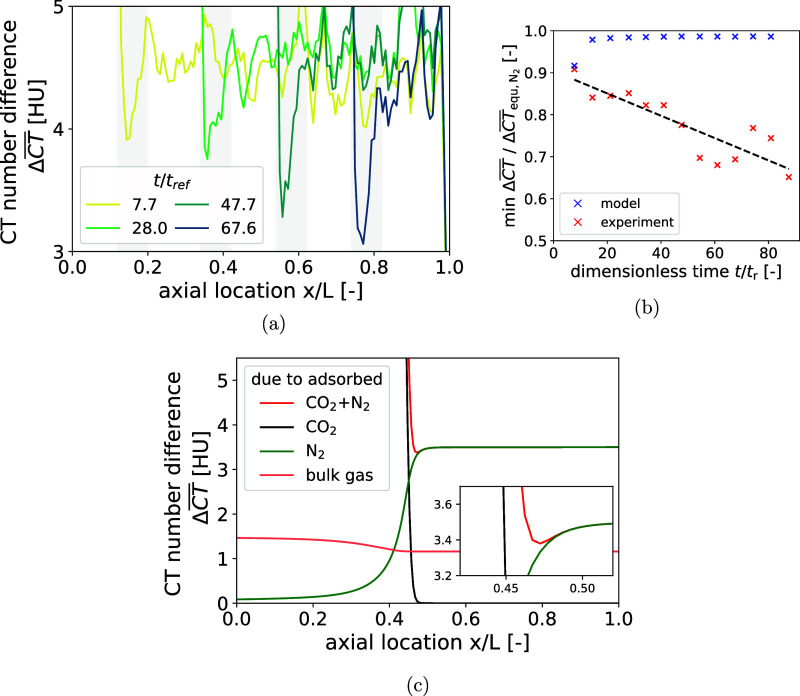
CT number difference profiles, $\Delta \overset{®}{CT}=\overset{®}{CT}\left(t,X;\theta \left(t,X\right)\right)-\overset{®}{CT}\left(X;\theta^{*}\right)$ , capturing a transient reduction in $\Delta \overset{®}{CT}$ (i.e., N_2_ desorption) occurring ahead of a second increase in $\Delta \overset{®}{CT}$ , which is associated with the initial CO_2_ adsorption during the binary CO_2_–N_2_ DCB experiment. (a) Experimental profiles at selected relative times (every third time point) to highlight the evolving dip. (b) Minimum value of the dip, averaged over a 3 mm slice at the local minimum, plotted relative to the plateau mean (experiment: 4.469 HU, model: 4.61 HU). (c) Model-reconstructed CT number difference profiles at *t*/*t*_ref_ = 34.4, showing the contributions of the two adsorbed components and bulk gas.

### Thermally Enhanced Nitrogen Roll-Up during Binary DCB

The present observation, a local reduction in CT number and thus adsorbed amount before the arrival of the strongly adsorbing component, is reminiscent of thermally influenced roll-up phenomena previously reported in the literature. Li et al. described a dual-mode roll-up in CO_2_–H_2_O systems, where a thermal wave detached from the main concentration front and propagated further ahead, producing early desorption of the weaker component.^19^ Pirngruber et al. observed a similar but more tightly coupled behavior for CO_2_–CH_4_ breakthrough experiments, in which the thermal wave advanced slightly ahead of, yet remained linked to, the equilibrium-displacement front.^20^ Ahn et al., in contrast, reported an enhancement of the classical roll-up—that is, thermal effects modifying the magnitude and shape of the equilibrium-driven roll-up without forming a distinct, preceding thermal wave.^21^ The behavior observed here most closely aligns with this latter case: a thermally assisted desorption of N_2_ that amplifies the classical single-mode roll-up rather than generating a fully separated thermal front.

This interpretation is supported by the experimental data. The reduction of the CT number ahead of the CO_2_ adsorption wave by 10% and 35% corresponds to adsorbed amounts of 0.1944 mol/kg and 0.1255 mol/kg, respectively, assuming the bulk gas phase is pure N_2_. Using the fitted dual-site Langmuir isotherm for N_2_ on zeolite 13X at atmospheric pressure, these reductions translate to local temperature increases of approximately 9 K (303.15 K) and 29.5 K (323.65 K), the latter of which coincides with the measured peak temperatures at the four thermocouples (328.1–324.4 K from first to fourth position). A closer inspection of the total flow-rate response curves (Figure 10) further supports this view. While the flow rate remains elevated between N_2_ and CO_2_ breakthroughs, reflecting the equilibrium-displacement-induced roll-up, a pronounced enhancement immediately following N_2_ breakthrough indicates an additional thermally driven contribution. Moreover, the small dip observed just before CO_2_ breakthrough corresponds to the arrival of the thermal front—and thus of the region already depleted in adsorbed N_2_—at the column outlet. Taken together, these observations indicate that the phenomenon corresponds to a thermally enhanced single-mode roll-up, rather than a fully detached dual-mode wave. It is also consistent with the observation that thermally induced desorption increases over the course of the experiment: as heat accumulates in the bed, more energy is transported downstream, enhancing desorption in the later regions of the column. However, these experimental findings contrast with the model predictions, which, while reproducing a roll-up, show only limited evidence of thermal enhancement. Notably, Wilkins et al.^14^ do not explicitly report the roll-up in terms of flow rate, as they found it experimentally difficult to measure, nor do they indicate observing a thermally enhanced roll-up. Their careful treatment of nonisothermal behavior should, in principle, capture such effects. Nevertheless, it must therefore be assumed that the heat effects are not fully captured by the model. While the temperature profile is generally well reproduced in magnitude, differences remain. The experiment shows a more gradual initial rise before steeper gradients, whereas the model predicts a more abrupt increase. Moreover, the poor fit of the N_2_ temperature wave suggests that the heat transfer coefficients may not be constant, but instead vary with local temperature and flow rate. While the collective interpretation of the experimental results, point toward the presence of a thermally enhanced roll-up, further experiments, for example with a bed initially saturated with CO_2_ at a lower concentration, which is then purged with a pure CO_2_ front could potentially provide clarity, as in this case the DA method could be used to accurately calculate the adsorbed amount, and prove the desorption of N_2_. Furthermore, it could be investigated what definition and parameters of the models would need to be modified to reproduce the thermally enhanced roll-up.

**Figure 10:**
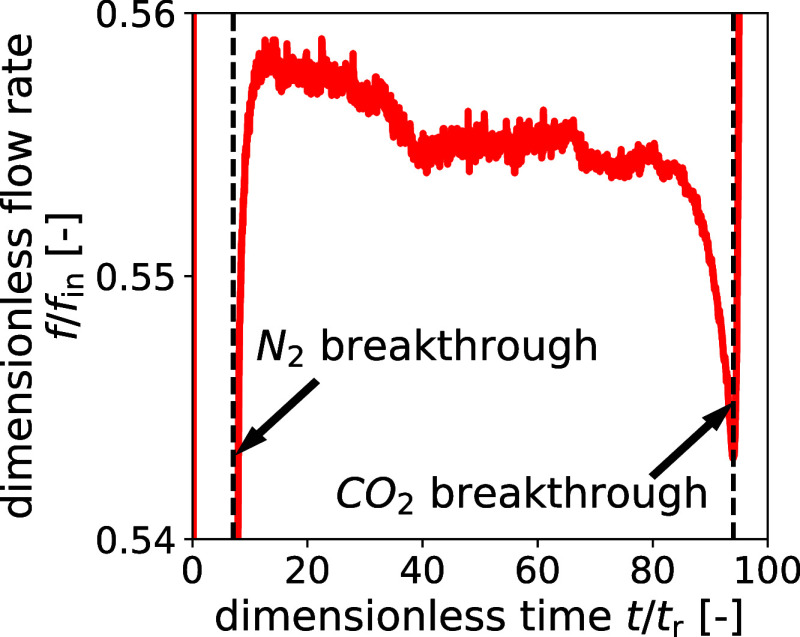
Breakthrough response (total flow rate) of the binary CO_2_–N_2_ DCB experiment, showing the enhanced roll-up phenomenon of the weakly adsorbing nitrogen.

## Conclusion

This study successfully extended the DA method, i.e., the augmentation of adsorption experiments with XCT, from unary systems with strongly adsorbing CO_2_ to unary and binary systems with weakly adsorbing N_2_. Using the DA method, adsorption isotherms of CO_2_ and N_2_ on commercial activated carbon and zeolite 13X were accurately measured, and the role of the bulk gas phase in interpreting CT number changes was quantified, demonstrating the necessity of fully accounting for it. A unary DCB experiment with N_2_ on zeolite 13X showed that, with a suitable correction factor, the DA method can reliably track the transient progression of the internal adsorbed phase under near-isothermal, equilibrium-controlled conditions. Limitations were encountered in a binary CO_2_–N_2_ system, where the adsorbed amount could not be directly determined without knowledge of the local adsorbed-phase composition; nonetheless, complementation with a one-dimensional DCB model highlighted that XCT can provide valuable insights into complex dynamic phenomena. Both fast N_2_ and slower CO_2_ adsorption stages were well captured, and a local reduction in CT number ahead of CO_2_ adsorption indicated a thermally enhanced roll-up, i.e., N_2_ desorption driven by propagated heat—a phenomenon rarely observed due to measurement challenges. These findings highlight that XCT can be meaningfully utilized to improve our understanding of complex dynamic adsorption processes and, in doing so, can inform the development of more accurate process models. Building on this capability, the present study lays the groundwork for extending the DA method to cyclic adsorption processes, where measurements of internal loading profiles could provide experimental access to phenomena that are currently accessible only through simulations.

## Supplementary Material



## Notes

Equations of the one-dimensional dynamic adsorption model; gas density–CT number calibration; X-ray noise estimation for gas density calibration and packed-bed measurements; uncertainty analysis; condition for the extremum of *R*(*p*); equilibrium adsorption isotherm data; additional temperature and internal profile data from dynamic column breakthrough experiments (PDF)
