# Supplementary material for: Unary and Binary Dynamic Column Breakthrough Experiments with Carbon Dioxide and Nitrogen Imaged by X‑ray Computed Tomography
Source: Langmuir. 2026 May 21;42(21):14716–32. doi: 10.1021/acs.langmuir.6c00250 (PMC13235649; doi:10.1021/acs.langmuir.6c00250)
Supplement: Supplementary file 1 [file la6c00250_si_001.pdf]

Unary and Binary Dynamic Column Breakthrough Experiments with Carbon Dioxide and Nitrogen imaged by X-ray Computed Tomography

David Büchner and Ronny Pini*

Department of Chemical Engineering, Imperial College London, London SW7 2AZ, United Kingdom

E-mail: r.pini@imperial.ac.uk

Content List

1. Equations of the 1-D Dynamic Adsorption Model
2. Gas density - CT number calibration
3. X-ray noise estimation - Gas density CT number calibration
4. X-ray noise estimation - Packed bed
5. Uncertainty considerations
6. Condition for the extremum of $R(p) = B(p)/A(p)$
7. Equilibrium adsorption isotherm data
8. Dynamic Column Breakthrough experiments

1. Equations of the 1-D Dynamic Adsorption Model

The 1-D model used to simulate the Dynamic Column breakthrough (DCB) experiments is composed of a set of partial differential equations (PDEs). The dimensional forms of the PDEs is summarized here.

Overall mass balance

$$\frac{1}{P}\frac{\partial P}{\partial t} - \frac{1}{T}\frac{\partial T}{\partial t} = -\frac{T}{P}\frac{\partial}{\partial z}\left(\frac{P}{T}\nu\right) - \frac{RT}{P}\frac{\rho_b}{\epsilon}\sum_{i=1}^{n_c}\frac{\partial q_i}{\partial t} \tag{1}$$

Component material balance for component i

$$\frac{\partial y_i}{\partial t} + \frac{y_i}{P}\frac{\partial P}{\partial t} - \frac{y_i}{T}\frac{\partial T}{\partial t} = \frac{T}{P}D_L\frac{\partial}{\partial z}\left(\frac{P}{T}\frac{\partial y_i}{\partial z}\right) - \frac{T}{P}\frac{\partial}{\partial z}\left(\frac{y_i P}{T}\nu\right) - \frac{RT}{P}\frac{\rho_b}{\epsilon}\frac{\partial q_i}{\partial t} \tag{2}$$

Solid phase mass balance

$$\frac{\partial q_i}{\partial t} = k_i\left(q_i^* - q_i\right), k_i = \frac{15\epsilon_p D_m}{\tau r_p^2} \tag{3}$$

Pressure drop

$$-\frac{\partial P}{\partial z} = \frac{150}{4}\frac{1}{r_p^2}\left(\frac{1-\epsilon}{\epsilon}\right)^2 \mu\nu \tag{4}$$

Column energy balance

$$\left[\frac{\rho_b}{\epsilon}\left(C_{p,s} + C_{p,a}\sum_{i=1}^{n_c} q_i\right)\right]\frac{\partial T}{\partial t} = \frac{K_z}{\epsilon}\frac{\partial^2 T}{\partial^2 z} - \frac{C_{p,g}}{R}\frac{\partial}{\partial z}(\nu P) - \frac{C_{p,g}}{R}\frac{\partial P}{\partial t} - \frac{\rho_b}{\epsilon}C_{p,a}\sum_{i=1}^{n_c}\frac{\partial q_i}{\partial t} + \frac{\rho_b}{\epsilon}\sum_{i=1}^{n_c}\left((-\Delta U_i + RT)\frac{\partial q_i}{\partial t}\right) - \frac{2h_{in}}{\epsilon r_{in}}(T - T_W) \tag{5}$$

Wall energy balance

$$\rho_W C_{p,W}\frac{\partial T_W}{\partial t} = K_W\frac{\partial^2 T_W}{\partial^2 z} + \frac{2r_{in}h_{in}}{r_{out}^2 - r_{in}^2}(T - T_W) - \frac{2r_{out}h_{out}}{r_{out}^2 - r_{in}^2}(T_W - T_\infty) \tag{6}$$

2. Gas density - CT number calibration

The calibration curve linking gas density to attenuation is defined through the linear relation

$$CT_{\mathrm{g}}(\rho) = a\rho + b. \tag{7}$$

For the three gases (He, CO_2, and N_2) the parameter a and b, where obtained through filling the empty column with the respective gas, varying the pressure thus density. The parameters were calculated over a 100 mm long section, removing the outer part of the void, that is, over a cylindrical section with a radius of 10 mm, total volume = 31 416 mm^3.

Figure 1 (a) shows the CT number over density for all three gases. The values mostly collapse on a single linear curve with slope $a = 1$ HU m^{3}kg-1. To allow for a more general description of the Digital Adsorption framework and the possibility to explicitly calculate mixture CT numbers through a mass weighted combination, all three gases were fitted individually using a simple least-square algorithm. The fitted curves are shown in Figure 1 (b) and the corresponding parameters are summarized in Table 1.

Table 1: Gas CT number calibration parameters

Parameter	a [HU m^3 kg^{-1}]	b [HU]
He	1.137 ± 0.044	-1009.20 ± 0.03
N_2	0.9221 ± 0.005	-1008.91 ± 0.02
CO_2	0.9744 ± 0.004	-1008.99 ± 0.03

3. X-ray noise estimation - Gas density CT number calibration

The uncertainty associated with the CT number of the pure gases was calculated as the standard deviation from the mean CT number, in the pure gas scans acquired for the gas density CT number calibration in the previous section. The uncertainty was calculated for

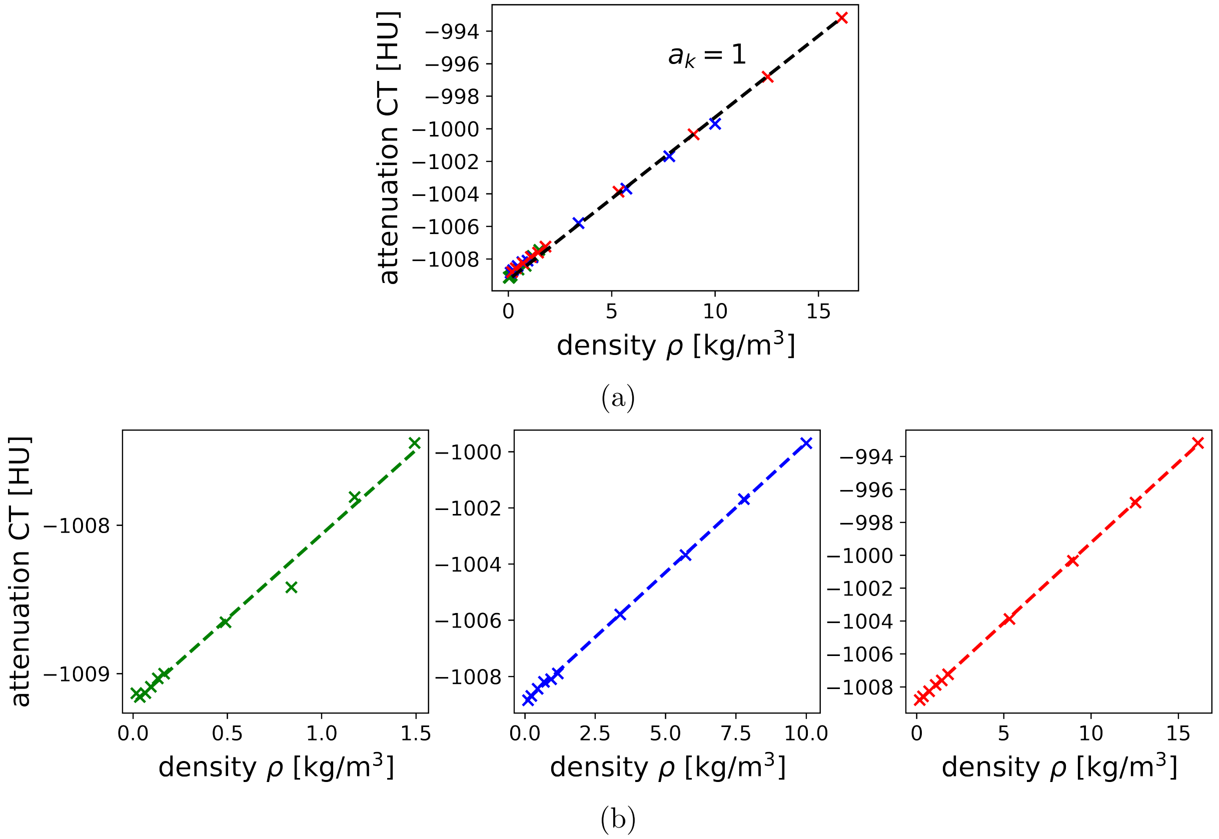

Figure 1: CT number over gas density, as measured with the medical CT scanner. Showing the collapsing of all three gas CT numbers onto a calibration line with a slope of $a = 1$ m^3 kg^{-1} (a), and the individual curves for all three gases (b): He left (green), N_2 middle (blue), and CO_2 right (red).

three different pressures, and eight different volume elements, for the large volumes the XCT scans was down sampled, i.e. averaged over multiple adjacent voxels. Figure 2 shows the uncertainties in the gas CT number for He, N_2, and CO_2, respectively. It is apparent that the

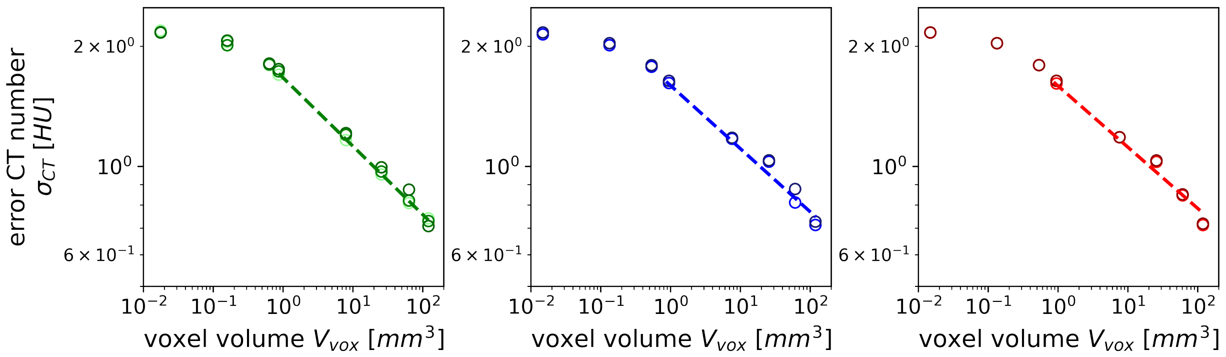

Figure 2: Uncertainty associated with CT number of the pure gases, as measured with the medical CT scanner, He left (green), N_2 middle (blue), and CO_2 right (red). Light green/blue/red: 0.1 bar, green/blue/red: 1 bar, and dark green/blue/red: 9 bar.

curves have two distinct sections: initially the uncertainty decreases at a lower but increasing rate towards large sampling volumes, until it converges to a curve characterized by a classical power low (constant slop in log-log plot). This is in line with, Pini et al.,[1] who for a packed bed observed a linear relationship between CT noise and the mean CT number, as well as between the logarithms of CT noise and voxel size. Above a certain voxel size, the CT noise can therefore be described by the following equation.

$$\sigma_{CT}\left(\overline{CT}, V_{vox}\right) = \left(c_1\overline{CT} + c_2\right) V_{vox}^{c_3} \tag{8}$$

Using a least square method the the parameter c_1, c_2, and c_3 are fitted to the data for all three gases individually, and summarized in Table 2. The small values of c_1 demonstrate

Table 2: Gas CT number calibration parameters

Parameter	c_1 [-]	c_2 [HU]	c_3 [-]
He	3.007×10^{-20}	1.650	-0.1652
N_2	1.604×10^{-21}	1.595	-0.1585
CO_2	2.908×10^{-21}	1.591	-0.1536

that the uncertainty of the CT number related to the mean CT number $\sigma_{CT_{gas}}$ is only weakly or not dependent on the pressure, i.e. the CT number. For gases, equation 8 can therefore be simplified to $\sigma_{CT}\left(V_{vox}\right) = c_2 V_{vox}^{c_3}$. As the CT numbers were obtained by averaging over a volume of 31 416 mm^3, the uncertainty associated with the CT number of the gas at atmospheric pressure is 0.2982 HU, 0.3090 HU and 0.3243 HU, for He, N_2, and CO_2, respectively.

4. X-ray noise estimation - Packed bed

Figure 3 shows the histograms of voxel-wise differences obtained from two repeated scans of the Zeolite 13X bed. The difference values are centered around zero and follow an approximately normal distribution. The width of the histogram directly reflects the intrinsic CT noise for the material under identical scan conditions, and it decreases with increasing voxel size through in-plane coarsening. Furthermore, when averaging N scans of the same slice prior to subtraction, the standard deviation of the difference image decreases approximately with $1/\sqrt{N}$, in agreement with classical error propagation principles. Figure 4 presents the measured standard deviation of CT numbers as a function of voxel volume for different materials (13X, AC, and PEEK). These standard deviations correspond to those derived from the histograms of voxel-wise differences shown in Figure 3. As expected, the CT noise decreases with increasing voxel volume due to spatial averaging. Two sampling approaches are distinct: in-slice coarsening (circles) and cuboidal resampling, including larger voxel volumes (crosses), which allow investigation of the effect of volumetric averaging on noise reduction. To quantitatively describe these trends, a CT-number–dependent noise model was fitted jointly to the 13X and AC datasets, using the same formulation introduced previously for the uncertainty quantification of the pure gas CT numbers. Using a least-squares algorithm, the model parameters were fitted to the data, yielding best-fit values of $c_1 = 5.047 \times 10^{-3}$, $c_2 = 13.71\,\text{HU}$, and $c_3 = -0.3483$.

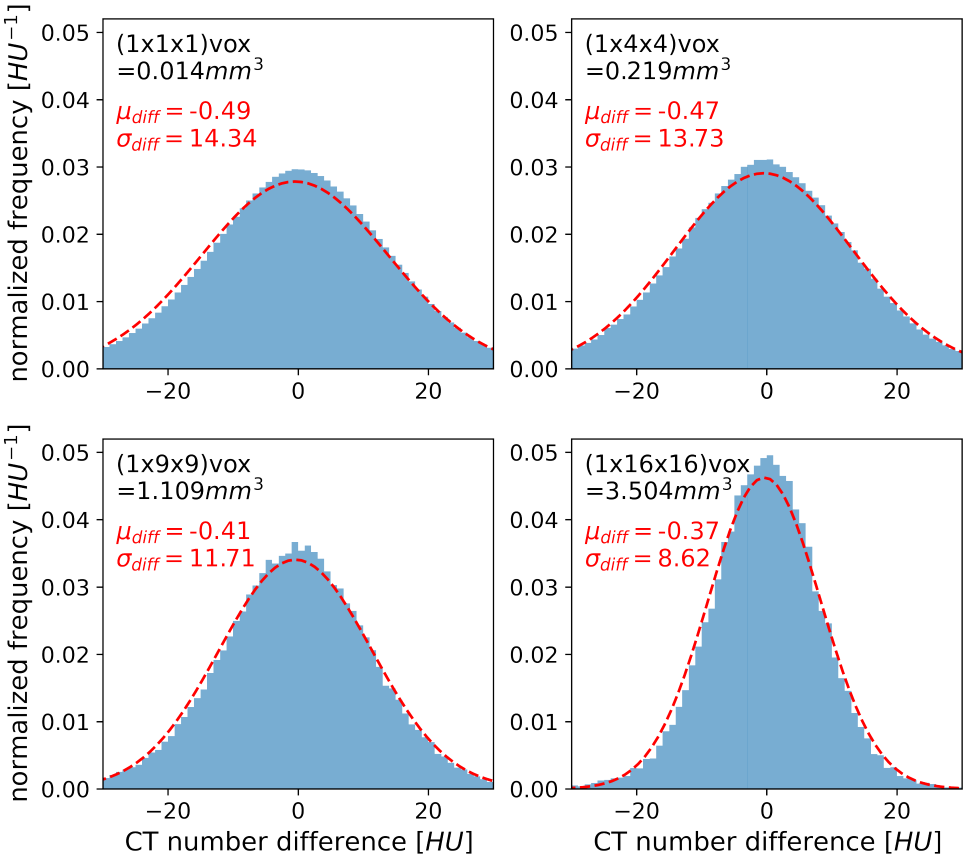

Figure 3: Histograms of difference images obtained from two sequential scans of the zeolite 13X bed. Tthe images were reconstructed using in-plane voxel coarsening within a single slice, with coarsening schemes of $1 \times 1 \times 1$, $1 \times 4 \times 4$, $1 \times 9 \times 9$, and $1 \times 16 \times 16$.

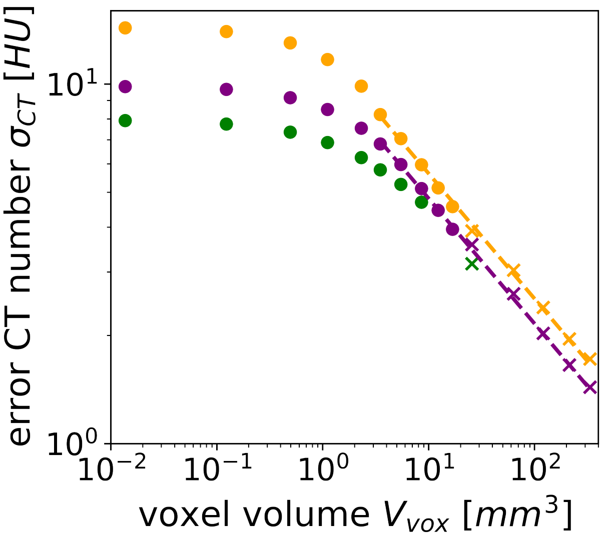

Figure 4: Error in CT number as a function of voxel volume for 13X (orange), AC (purple), and PEEK (green). Circles represent in-slice resampling with 1 mm slice thickness, while crosses correspond to cuboidal voxel volumes, including larger volumetric averaging.

Here, c_1 scales the noise contribution with the CT number, c_2 represents a baseline reference level, and c_3 captures the voxel-volume dependence. Both materials were included in a single fit to characterize the general behavior of X-ray noise across different attenuation levels. Nevertheless, deviations between 13X and AC remain visible due to their significantly different mean CT numbers. This highlights the importance of including the $\overline{CT}$-dependence in Eq. 8, ensuring that the noise–volume relationship can be consistently applied across different materials.

5. Uncertainty considerations

In this section, we describe the uncertainty analysis performed for the bed properties used in our experiments. First-order (linear) uncertainty propagation was applied to the geometrical measurements, mass, and derived quantities such as bulk density, porosity, thermal conductivity, and viscosity. The method assumes uncorrelated input uncertainties and small relative errors, following the standard approach outlined by Taylor.[2]

The volume of a cylindrical bed is defined as

$$V_{\rm bed} = \frac{\pi}{4} D^2 L, \tag{9}$$

where D and L are the diameter and length of the cylinder, respectively. Using first-order (linear) uncertainty propagation for uncorrelated input uncertainties,[2] the standard deviation of the bed volume is

$$\sigma_{\rm V_{bed}} = \sqrt{\left(\frac{\partial V}{\partial D}\sigma_{\rm D}\right)^2 + \left(\frac{\partial V}{\partial L}\sigma_{\rm L}\right)^2} = \sqrt{\left(\frac{\pi}{2} DL\,\sigma_{\rm D}\right)^2 + \left(\frac{\pi}{4} D^2\,\sigma_{\rm L}\right)^2}.$$

with partial derivatives

$$\frac{\partial V_{\rm bed}}{\partial D} = \frac{\pi}{2} DL, \quad \frac{\partial V_{\rm bed}}{\partial L} = \frac{\pi}{4} D^2. \tag{10}$$

For the dimensions $D = 0.030\,\mathrm{m}$, $L = 0.273\,\mathrm{m}$ and machining uncertainties $\sigma_{\rm D} = 1 \times 10^{-4}\,\mathrm{m}$, $\sigma_{\rm L} = 2 \times 10^{-4}\,\mathrm{m}$, this yields

$$V_{\rm bed} = 1.92972 \times 10^{-4}\,\mathrm{m}^3, \quad \sigma_{\rm V_{bed}} \approx 1.29 \times 10^{-6}\,\mathrm{m}^3. \tag{11}$$

The bed (bulk) density is defined as

$$\rho_{\rm bed} = \frac{m_{\rm bed}}{V_{\rm bed}}, \tag{12}$$

where $m_{\rm bed}$ is the measured mass of the bed. Using first-order propagation for a quotient, assuming uncorrelated uncertainties in mass and volume, the uncertainty in $\rho_{\rm bed}$ is

$$\sigma_{\rho_{\rm bed}} = \rho_{\rm bed}\sqrt{\left(\frac{\sigma_{\rm m_{bed}}}{m_{\rm bed}}\right)^2 + \left(\frac{\sigma_{\rm V_{bed}}}{V_{\rm bed}}\right)^2}. \tag{13}$$

The total porosity is computed as

$$\Phi_{\mathrm{tot}} = 1 - \frac{\rho_{\mathrm{bed}}}{\rho_{\mathrm{s}}}, \tag{14}$$

where ρ_{s} is the solid (particle) density. First-order uncertainty propagation then gives

$$\sigma_{\Phi_{\mathrm{tot}}} = \sqrt{\left(\frac{\sigma_{\rho_{\mathrm{bed}}}}{\rho_{\mathrm{s}}}\right)^2 + \left(\frac{\rho_{\mathrm{bed}}\,\sigma_{\rho_{\mathrm{s}}}}{\rho_{\mathrm{s}}^2}\right)^2}. \tag{15}$$

This can be written in a relative form as

$$\sigma_{\Phi_{\mathrm{tot}}} \approx \Phi_{\mathrm{tot}}\sqrt{\left(\frac{\sigma_{\rho_{\mathrm{bed}}}}{\rho_{\mathrm{bed}}}\right)^2 + \left(\frac{\sigma_{\rho_{\mathrm{s}}}}{\rho_{\mathrm{s}}}\right)^2}. \tag{16}$$

only when using the small-error approximation and after algebraic rearrangement; the form above with explicit ρ_{s} and ρ_{bed} is algebraically exact to first order.

According to[3] the uncertainty in the CT number related to the skeletal density of the adsorbent material $\overline{CT}_{\mathrm{s}}$ is calculated as

$$\sigma_{\mathrm{CT_s}} = \sqrt{\left(\frac{\sigma_{\overline{\mathrm{CT}}_{\mathrm{i}}}}{1-\Phi_{\mathrm{tot}}}\right)^2 + \left(\frac{\sigma_{\mathrm{CT_i}}}{1-\Phi_{\mathrm{tot}}}\right)^2 + \left(\frac{\left(\overline{CT}_{\mathrm{i}} - CT_{\mathrm{i}}\right)\sigma_{\Phi_{\mathrm{tot}}}}{(1-\Phi_{\mathrm{tot}})^2}\right)^2}. \tag{17}$$

First-order uncertainty propagation then gives the definition of the uncertainty in the excess CT number H^{ex}

$$\sigma^2_{\mathrm{H^{ex}}} = \sigma^2_{\overline{\mathrm{CT}}_{\mathrm{a}}} + \sigma^2_{\overline{\mathrm{CT}}_{\mathrm{i}}} + \sigma^2_{\Phi_{\mathrm{tot}}}\left(CT_{\mathrm{a}} - CT_{\mathrm{i}}\right)^2 + \left(\sigma^2_{\mathrm{CT_a}} - \sigma^2_{\mathrm{CT_i}}\right)\Phi^2_{\mathrm{tot}} \tag{18}$$

and subsequently in the excess adsorbed amount

$$\sigma_{\eta_{\mathrm{V}}^{\mathrm{ex}}} = \eta_{\mathrm{V}}^{\mathrm{ex}}\sqrt{\left(\frac{\sigma_{\mathrm{H^{ex}}}}{H^{ex}}\right)^2 + \left(\frac{\sigma_{\mathrm{a}}}{a}\right)^2 + \left(\frac{\sigma_{\rho_{\mathrm{bed}}}}{\rho_{\mathrm{bed}}}\right)^2}. \tag{19}$$

6. Condition for the extremum of $R(p) = B(p)/A(p)$

We consider

$$R(p) = \frac{B(p)}{A(p)} \tag{20}$$

with

$$A(p) = \overline{CT}(p_{\mathrm{eq}}) - \overline{CT}(p^*), \qquad B(p) = -\phi_{\mathrm{tot}}\,[CT_{\mathrm{g}}(p_{\mathrm{eq}}) - CT_{\mathrm{g}}(p^*)]. \tag{21}$$

Step 1: General condition for the extremum

Differentiating $R(p)$ gives

$$\frac{dR}{dp} = \frac{A\,\dfrac{dB}{dp} - B\,\dfrac{dA}{dp}}{A^2}. \tag{22}$$

The stationary points of $R(p)$ must satisfy $\frac{dR}{dp} = 0$ thus

$$A(p)\frac{dB}{dp} = B(p)\frac{dA}{dp}. \tag{23}$$

Dividing by $A(p)B(p)$ (for $A \neq 0$, $B \neq 0$) leads to the compact logarithmic condition

$$\boxed{\frac{d}{dp}\ln A(p) = \frac{1}{B(p)}\frac{dB}{dp}} \tag{24}$$

which holds independently of the explicit forms of $A(p)$ and $B(p)$.

Step 2: Explicit form of $B(p)$

Using the linear model for the bulk-gas CT number,

$$CT_{\mathrm{g}}(p) = \frac{a}{R_{\mathrm{sp}}T}\,p + b, \tag{25}$$

and treating $CT_{\mathrm{g}}(p^*)$ and ϕ_{tot} as constants, we obtain

$$B(p) = -\phi_{\mathrm{tot}}\left(\frac{a}{R_{\mathrm{sp}}T}\,p + b - CT_{\mathrm{ref}}\right), \qquad \frac{dB}{dp} = -\phi_{\mathrm{tot}}\frac{a}{R_{\mathrm{sp}}T}. \tag{26}$$

Substituting into (24) yields the explicit condition

$$\boxed{\frac{d}{dp}\ln\!\big(\overline{CT}(p_{\mathrm{eq}}) - \overline{CT}(p^*)\big) = \frac{\dfrac{a}{R_{\mathrm{sp}}T}}{\dfrac{a}{R_{\mathrm{sp}}T}p + b - CT_{\mathrm{g}}(p^*)}} \tag{27}$$

which implicitly determines the pressure $p^{\min}$ at which $R(p)$ attains a stationary point. Here, the right-hand side is an explicit function of p containing only known physical constants, while the left-hand side can be obtained directly from the measured $\overline{CT}(p_{\mathrm{eq}})$.

Step 3: Explicit form of $\eta_{\mathrm{ex}}(p)$

To connect the condition to adsorption isotherm models, we approximate the excess CT contribution by a dual-site Langmuir (DSL) form,

$$\eta_{\mathrm{ex}}(p) = \frac{A(p) - B(p)}{a\rho_{\mathrm{b}}M_{\mathrm{m}}} \approx \frac{q_{\mathrm{s}1}b_1c}{1 + b_1c} + \frac{q_{\mathrm{s}2}b_2c}{1 + b_2c}, \qquad c = \frac{p}{RT}. \tag{28}$$

Then

$$A(p) = B(p) + a\rho_{\mathrm{b}}M_{\mathrm{m}}\eta_{\mathrm{ex}}(p), \tag{29}$$

so the stationary condition becomes

$$\frac{d}{dp}\ln\!\big(B(p) + a\rho_{\mathrm{b}}M_{\mathrm{m}}\eta_{\mathrm{ex}}(p)\big) = \frac{1}{B(p)}\frac{dB}{dp}. \tag{30}$$

Expanding explicitly gives

$$\frac{\frac{dB}{dp} + a\rho_{\mathrm{b}} M_{\mathrm{m}} \frac{d\eta_{\mathrm{ex}}(p)}{dp}}{B(p) + a\rho_{\mathrm{b}} M_{\mathrm{m}} \eta_{\mathrm{ex}}(p)} = \frac{1}{B(p)} \frac{dB}{dp}, \tag{31}$$

where

$$\frac{d\eta_{\mathrm{ex}}(p)}{dp} = \frac{q_{\mathrm{s1}} b_1 RT}{(RT + b_1 p)^2} + \frac{q_{\mathrm{s2}} b_2 RT}{(RT + b_2 p)^2}. \tag{32}$$

and

$$B(p) = -\phi_{\mathrm{tot}} \left(\frac{a}{R_{\mathrm{sp}} T} p + b - CT_{\mathrm{ref}} \right), \quad \frac{dB}{dp} = -\phi_{\mathrm{tot}} \frac{a}{R_{\mathrm{sp}} T}. \tag{33}$$

Thus, the condition for the extremum of $R(p)$ becomes the fully explicit equation

$$\boxed{\frac{-\phi_{\mathrm{tot}} \frac{a}{R_{\mathrm{sp}} T} + \frac{a\rho_{\mathrm{b}} M_{\mathrm{m}}}{RT} \left(\frac{q_{\mathrm{s1}} b_1}{\left(1 + b_1 \frac{p}{RT}\right)^2} + \frac{q_{\mathrm{s2}} b_2}{\left(1 + b_2 \frac{p}{RT}\right)^2} \right)}{-\phi_{\mathrm{tot}} \left(\frac{a}{R_{\mathrm{sp}} T} p + b - CT_{\mathrm{ref}} \right) + a\rho_{\mathrm{b}} M_{\mathrm{m}} \left(\frac{q_{\mathrm{s1}} b_1 \frac{p}{RT}}{1 + b_1 \frac{p}{RT}} + \frac{q_{\mathrm{s2}} b_2 \frac{p}{RT}}{1 + b_2 \frac{p}{RT}} \right)} = \frac{\frac{a}{R_{\mathrm{sp}} T}}{\frac{a}{R_{\mathrm{sp}} T} p + b - CT_{\mathrm{ref}}}} \tag{34}$$

which can be solved numerically for p_{min}, yielding the exact pressure at which the relative contribution of the bulk gas to the total CT change reaches a minimum.

7. Equilibrium adsorption isotherm data

The adsorption isotherm data as measured with the Digital Adsorption method (at 294.15 K) is reported for CO_2 and N_2 for zeolite 13X and activate carbon in Table 3 and 4, respectively.

Table 3: Equilibrium adsorption isotherms of Zeolite 13X

CO_2		N_2	
pressure p [Pa]	excess amount η^{ex} [mol/kg]	pressure p [Pa]	excess amount η^{ex} [mol/kg]
10500	2.797 ± 0.0355	9600	0.0272 ± 0.0141
20600	3.160 ± 0.0395	19900	0.0547 ± 0.0141
38400	3.637 ± 0.0447	40500	0.1042 ± 0.0141
56200	3.940 ± 0.0481	60600	0.1602 ± 0.0142
79600	4.133 ± 0.0502	76900	0.1943 ± 0.0143
101100	4.401 ± 0.0532	101200	0.2585 ± 0.0144
107000	4.266 ± 0.0517	100500	0.2458 ± 0.0144
215300	4.732 ± 0.057	203100	0.4687 ± 0.0151
254300	4.758 ± 0.0573	310100	0.6256 ± 0.016
501000	5.034 ± 0.0606	502400	0.8949 ± 0.0178
688000	5.184 ± 0.0624	701700	1.109 ± 0.0195
871200	5.255 ± 0.0634	897500	1.284 ± 0.0212

Table 4: Equilibrium adsorption isotherms of Activated carbon Norit RB3

CO_2		N_2	
pressure p [Pa]	excess amount η^{ex} [mol/kg]	pressure p [Pa]	excess amount η^{ex} [mol/kg]
9200	0.571 ± 0.0188	10400	0.0297±0.0273
20300	0.903 ± 0.0213	21200	0.0713±0.0274
41600	1.392 ± 0.0264	40100	0.155±0.0279
60900	1.75 ± 0.0308	59600	0.2287±0.0285
80400	2.097 ± 0.0354	77100	0.2999±0.0292
102900	2.396 ± 0.0397	103000	0.4069±0.0306
103800	2.272 ± 0.0382	102100	0.3901±0.0305
206700	3.422 ± 0.0565		
277400	3.927 ± 0.0661	292100	0.876±0.0454
485400	5.054 ± 0.0919	491600	1.224±0.0651
708900	5.767 ± 0.1159	710100	1.504±0.0872
856200	6.266 ± 0.1326	883200	1.763±0.1073

8. Dynamic Column Breakthrough experiments

Fitting of heat transfer coefficients

The inside and outside heat transfer coefficients of the 1-D DCB model were fitted against the temperature data from the experiments. For the two unary experiments a single good fit could be achieved. However, in case of the two staged process of the binary CO_2-N_2 experiment it can be clearly seen in Figure 5, that the fitted model does not accurately predict the temperature increase due to N_2 adsorption. This can be explained by the fact that the

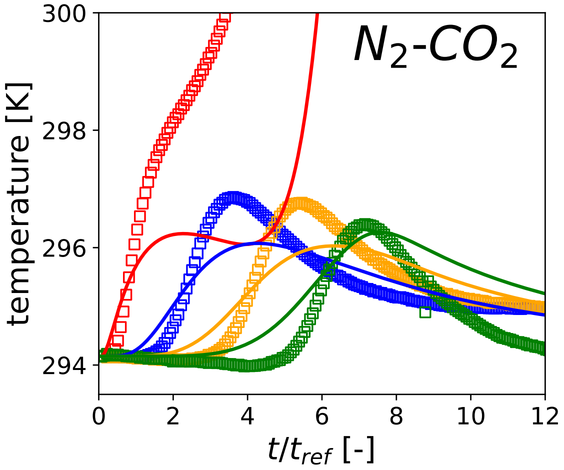

Figure 5: Temperature measurements of the binary CO_2–N_2 DCB experiment. The axis section was decreased to highlight the deviation between model prediction and experimental measurements during the N_2 adsorption phase.

breakthrough time of CO_2 is roughly an order of magnitude larger than that of N_2. Since each time step is weighted equally during the fitting process, the results are dominated by CO_2 adsorption, leading to significantly larger and longer-lasting temperature deviations. While this imbalance could be partially corrected by applying a different weighting scheme—such as giving each stage equal weight—it also underscores the general challenge of fitting a single constant heat transfer coefficient, which in reality depends on local and instantaneous conditions.

Correction factor method

The introduced correction factor method reformulates the contribution of the bulk gas phase to

$$\phi_{\mathrm{tot}}(X)\left[CT_{\mathrm{g}}(\theta(t,X)) - CT_{\mathrm{g}}(\theta^*)\right] \approx S(t,X)\phi_{\mathrm{tot}}(X)\left[CT_{\mathrm{g}}(\theta_{\mathrm{eq}}) - CT_{\mathrm{g}}(\theta^*)\right]. \tag{35}$$

This assumes that, at any given time and location, the adsorbed phase is in equilibrium with the bulk gas phase. In other words, the adsorption front travels at the same velocity and exhibits the same dispersion as the bulk gas phase. Figure 6 shows, for two different times as predicted by the model, the relative amount adsorbed, that is, the amount adsorbed relative to the amount expected if the bed were fully equilibrated and complete breakthrough had been achieved. The figure also shows the model predicted gas phase mole fraction. The

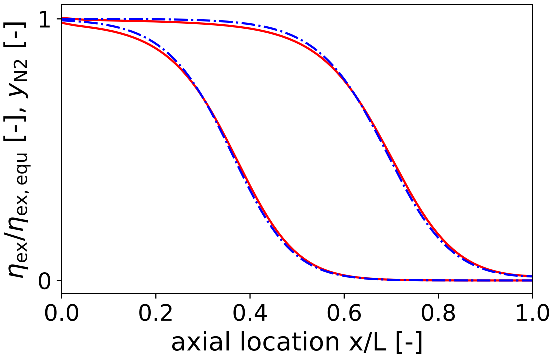

Figure 6: Internal profiles of the relative amount adsorbed $\eta^{\mathrm{ex}}/\eta^{\mathrm{ex,eq}}$ with $\eta^{\mathrm{ex,equ}} =$ 0.2358 mol/kg (red), and the mole fraction of nitrogen y_{N_2} (blue) for two discrete times $t/t_{ref} = 1.32$ and 2.54. Both were obtained from the fitted DCB model, and demonstrate the near perfect overlap.

near perfect overlap of both confirms the validity of the assumption that the adsorbed phase and the bulk gas phase are in equilibrium. In other words, the experiment is equilibrium controlled, and the correction factor method is therefore applicable.

References

References

(1) Joss, L.; Pini, R. Digital Adsorption: 3D Imaging of Gas Adsorption Isotherms by X-ray Computed Tomography. *Journal of Physical Chemistry C* **2017**, *121*, 26903–26915.

(2) Taylor, J. R.; Thompson, W. An Introduction to Error Analysis: The Study of Uncertainties in Physical Measurements. *Physics today* **1998**, *51*, 57–58.

(3) Pini, R. Interpretation of net and excess adsorption isotherms in microporous adsorbents. *Microporous and Mesoporous Materials* **2014**, *187*, 40–52.
